# Thrombomodulin facilitates melanoma progression via FAK- and ezrin-mediated phenotypic plasticity

**DOI:** 10.1186/s12929-026-01217-2

**Published:** 2026-01-27

**Authors:** Cheng-Hsiang Kuo, Ru-Han Sie, Ya-Chu Ku, Cheng-Lin Wu, Chao-Kai Hsu, Chao-Han Lai, Hua-Lin Wu

**Affiliations:** 1 Department of Physiology, College of Medicine, National Cheng Kung University, No.1, University Road, Tainan City, 701 Taiwan; 2 International Center for Wound Repair and Regeneration, National Cheng Kung University, Tainan, Taiwan; 3 Department of Biochemistry and Molecular Biology, College of Medicine, National Cheng Kung University, No.1, University Road, Tainan City, 701 Taiwan; 4 Department of Pathology, College of Medicine, National Cheng Kung University Hospital, National Cheng Kung University, Tainan, Taiwan; 5 Department of Dermatology, College of Medicine, National Cheng Kung University Hospital, National Cheng Kung University, Tainan, Taiwan; 6 Department of Surgery, College of Medicine, National Cheng Kung University Hospital, National Cheng Kung University, Tainan, Taiwan

**Keywords:** Melanoma, Vascular mimicry, Thrombomodulin, Plasticity, Ezrin, FAK

## Abstract

**Background:**

Cancer cell plasticity enables dynamic transitions between cellular states, contributing to tumor progression and the acquisition of phenotypic traits such as vascular mimicry (VM), which promotes malignancy and resistance to anti-angiogenic therapies. Thrombomodulin (TM), a type I transmembrane glycoprotein known for initiating sprouting angiogenesis, has been implicated in tumor vascularization. However, its role in melanoma progression and VM remains poorly characterized.

**Methods:**

TM expression was evaluated in human cutaneous melanoma biopsies and an endothelial–melanoma co-culture system. Functional assays were conducted to assess the impact of TM knockdown and overexpression on cell adhesion and VM formation. Domain-specific contributions of TM were investigated using constructs targeting its lectin-like domain and ezrin-binding motif. Mechanistic studies involved pharmacological inhibition of focal adhesion kinase (FAK) and siRNA-mediated silencing of ezrin. Therapeutic potential was assessed using a soluble TM lectin domain in both in vitro and in vivo melanoma models.

**Results:**

TM was expressed in both angiogenic and non-angiogenic vessels within melanoma tissues and co-culture systems. TM knockdown impaired cell adhesion and suppressed VM formation, while TM overexpression in TM-null melanoma cells enhanced cellular plasticity via its lectin-like domain and ezrin-binding motif. Inhibition of FAK or silencing of ezrin reversed the TM-induced phenotypic switch. Treatment with a soluble TM lectin domain reduced cancer cell plasticity in vitro and significantly inhibited melanoma tumor growth and metastasis in vivo.

**Conclusions:**

TM promotes melanoma cell plasticity and VM through FAK- and ezrin-dependent pathways. These findings position TM as a key regulator of tumor progression and suggest that targeting TM may offer a novel therapeutic strategy to disrupt cancer cell plasticity and suppress melanoma growth.

**Supplementary Information:**

The online version contains supplementary material available at 10.1186/s12929-026-01217-2.

## Background

Cell plasticity is essential for tissue homeostasis but contributes to tumorigenesis [1]. In particular, phenotypic plasticity in cancer, fueled by genetic or nongenetic mechanisms and tumor microenvironment, promotes cancer progression and therapy resistance [2, 3]. Non-angiogenic vascularization of tumors, such as vascular mimicry (VM), has been recognized as a major cause of resistance to anti-angiogenic therapy [4]. It is the formation of tube-like structures independent of vascular endothelial cells [5]. Thus, tumor cells can increase tumor blood perfusions through both angiogenesis and VM [6]. Targeting tumor vasculature through antiangiogenic agents has emerged as a promising strategy to suppress tumor growth [7]. However, this approach faces significant limitations, as tumors may circumvent vascular blockade by upregulating VM activity to sustain blood supply. Consequently, dual inhibition of angiogenesis and VM may offer a more effective strategy for disrupting tumor perfusion. While VM is strongly associated with melanoma invasiveness, aggressiveness, and metastatic potential [8], its underlying molecular mechanisms remain poorly understood compared to the well-characterized pathways governing angiogenesis. Further investigation is therefore warranted to elucidate the regulatory networks driving VM and to identify potential therapeutic targets.

Thrombomodulin (TM) is a multidomain type I transmembrane glycoprotein belonging to the C-type lectin family XIV members with diverse functions in vascular biology, inflammation, and cancer [9]. Structure homology and experimental analyses reveal the similarity among their molecular domains and functions in this protein family. In this regard, CD248, one of this protein family, promotes VM progression [10]. Structurally, mature TM consists of 5 distinct functional domains [11]. From the NH_2_-terminus is a C-type lectin-like domain. It is essential for TM-mediated cell adhesions [12]. Following is a region with six tandem epidermal growth factor-like repeats connected to a region enriched in serine/threonine. Then, a transmembrane domain is followed by a short cytoplasmic domain that bridges F-actin via ezrin [13]. Recent studies have revealed intricate intermolecular interactions of TM domains with other molecules, significantly influencing the overall functions of TM. Most notably, in endothelial cells, the association of the TM cytoplasmic domain with F-actin at the cell membrane mediates 3D podosome formation, initiating sprouting angiogenesis [14]. Thus, TM has been recognized as a key molecule mediating endothelial invasion during angiogenesis [15]. On the other hand, soluble recombinant TM lectin-like domain (rTMD1) inhibits angiogenesis, in part, via interfering with EGFR signaling through interaction with Lewis Y antigen [16]. TM enhances collective migration in epithelial cells through interactions between its cytoplasmic domain and the actin-binding protein, ezrin [13]. Under vascular remodeling, TM expression is upregulated in vascular smooth muscle cells, where it promotes cell migration through a focal adhesion kinase (FAK)-dependent mechanism [17]. In line with this finding, endogenous TM enhances the activation of plasminogen to become plasmin at the cell membrane surface, especially at lipid rafts and lamellipodia, thereby promoting pericellular proteolysis and cell migration during angiogenesis [18]. Furthermore, endothelial TM contributes to tumor angiogenesis by binding to fibronectin and activating FAK during cell adhesion [19]. Additionally, our recent study using a 3D melanoma spheroid model demonstrates that the TM-plasminogen system facilitates tumor invasion [20]. Although the mechanisms are not completely delineated, these findings support the notion that TM is critical in promoting cell adhesion and migration.

TM exerts diverse cellular functions that contribute to both protective and tumor-promoting roles, depending on the tumor type and cellular context [21, 22]. TM expression is a favorable factor in pancreatic cancer [23]. Whereas, higher expression of TM in soft tissue sarcomas is associated with highly recurrent, metastatic potential, and poor prognosis [24]. Though genetic and epigenetic regulation affecting TM expression in human melanoma cells [25] and the effect of its expression on cell migration and growth have been suggested [19, 26, 27], the molecular mechanism underlying TM in the malignancy of melanoma progression remains elusive. Given TM’s critical role in endothelial cell biology, particularly in regulating angiogenesis, we hypothesized that TM expression in melanoma may enhance cancer cell plasticity, with a specific impact on their capacity for VM. The current study, utilizing bioinformatic analysis, human skin biopsy, in vitro cell models, and in vivo mouse models of tumor growth and metastasis, investigated the biological functions of melanoma TM in cancer plasticity and demonstrated the potential application of soluble TM as decoy molecules against tumor progression in vitro and in vivo. The gain- and loss-of-function strategies and pharmaceutical suppression of FAK activity were conducted to delineate the underlying molecular mechanism of melanoma TM in VM activity. Thus, this study advances our understanding of the molecular mechanisms by which TM facilitates VM in melanoma and highlights TM as a potential therapeutic target for limiting melanoma progression.

## Methods

### Survival analysis

We used the Kaplan–Meier plotter database (http://kmplot.com/analysis/) to conduct a survival analysis of the THBD gene, the gene symbol of TM. This web-based tool analyzes survival analysis using different datasets from GEO, EGA, and TCGA repositories [28]. We conducted the survival analysis on default settings and the hazard ratio with 95% confidence intervals. Additionally, the survival analysis of the TM and its associated molecules in melanoma patients was conducted using the GEPIA2 online platform [29].

### Human skin biopsy

The human skin samples, including melanocytic nevus and melanoma tissue, were obtained during the surgical operation. The Institutional Review Board at the authors’ university hospital approved the study (A-ER-108-539).

### Reagents

The rTMD1 and AAV-TMD1 were prepared as previously described [16]. Antibodies used in this study are listed in the supplement Table S1.

### Cell culture and transfection

The human melanoma cells, including MeWo cells (RRID: CVCL_0445) and A2058 cells (RRID: CVCL_1059), were purchased from Bioresource Collection and Research Center (BCRB, Taiwan; originally from American Type Culture Collection, USA) and maintained in Minimum Essential Media and Dulbecco's modified Eagle's medium supplemented with 10% fetal bovine serum (FBS), respectively. The siRNA targeting human TM (SMARTpool), ezrin (SMARTpool), or scrambled siRNA control was purchased from GE Dharmacon. Manipulation of TM expression either by expression plasmid or siRNAs was performed using the Neon Transfection System (Invitrogen). The GFP-tagged human TM expression plasmids encoding full-length TM (TMG), lectin-like domain-deleted TM (TM-LeD) [12], and ezrin-binding motif mutation of TM cytoplasmic domain (TM-^522^RKK^524^/AAA, TM3A) gene [13] were included in this study, and the nucleotide sequence of each expression vector was confirmed by DNA sequencing. The pEGFP/N1 alone plasmid was used as a control.

### Western blot

Total proteins were analyzed under reduced and denatured conditions. The total protein was separated using SDS-PAGE and subsequently transferred to PVDF membranes. After blocking with non-fat milk, specific primary antibodies were applied, followed by horseradish peroxidase-conjugated secondary antibodies after washing with PBS supplemented with Tween-20. The signal of protein expression was then visualized using the enhanced chemiluminescence reagent (Pierce, USA) and analyzed using the iBright 1500 imaging system (Thermo Fisher Scientific, USA).

### Cell adhesion assay

The cells were allowed to adhere to laminin (0.1 mg/mL)- or Matrigel (0.9 mg/mL)-coated surfaces for the indicated periods (15 or 30 min). Then cells were fixed and stained for microfilaments using Alexa Fluor633-conjugated phalloidin to analyze the cell spreading area. The relative cell spreading area was measured using Image J software. To analyze focal adhesion signaling, cells were allowed to adhere to laminin- or Matrigel-coated surfaces for the indicated periods (0, 15, or 30 min). Then, the total cell lysates were collected for western blot analysis.

### VM activity assay

The VM activity assay was performed in 15-well μ-slides (ibidi GmbH, Planegg, Martinsried, Germany) coated with 10 μL Matrigel. Cells were washed with phosphate-buffered saline (PBS) and detached by HyQtase (GE Healthcare Life Sciences) cell detachment solution treatment followed by neutralization with an FBS-containing medium. MeWo cells, at 10,000 cells/well in medium containing 0.1% FBS, were applied on top of the Matrigel. A2058 cells at 5000 cells/well in medium containing 0.5% FBS were applied on top of the Matrigel. Cellular network formation on Matrigel was visualized at the indicated time points using an inverted microscope system (Leica DMI6000 B or Olympus IX71). The Image J software was utilized to measure the overall tube length. The VM activity was normalized and calculated relative to the total tube length**.**

### Immunofluorescence staining and laser-scanning confocal microscopy

For immunofluorescence staining, cells were cultured on laminin- or Matrigel-coated glass coverslips or on Matrigel in the μ-slide for the indicated periods. The cells were fixed with 2% paraformaldehyde in PBS at 37 °C for 30 min, followed by blocking and incubation with specific antibodies in PBS containing 0.5% bovine serum albumin and 0.25% Triton X-100. Coverslips were mounted with Anti-Fade Fluoromount G (Electron Microscopy Sciences). They were analyzed under an inverted microscope (Leica DMI6000 B) equipped with a digital EMCCD camera (iXon 897, Andor, Belfast, UK) and an N PLAN 10x/0.25, an HCX PL FLUOTAR L 20x/0.4, and an N PLAN APO 40x/0.8 objective lens at 25 °C. Colocalization of molecules was analyzed under a laser-scanning confocal microscope (FV3000, Olympus, Shinjuku-ku, Tokyo, Japan) equipped with a 20 × objective lens, a LUCPlanFL N 60x/0.7 objective lens, a UPLANSAPO 60x/1.35 oil objective lens, and a UPLANSAPO 100x/1.4 oil objective lens at 25 °C. To analyze TM expression in the co-culture of endothelial cells and melanoma cells, calcein AM-labeled MeWo cells and HUVECs were mixed according to a 10:1 ratio. The mixed cells were cultured on Matrigel for 4 h, followed by staining for TM and microfilaments. The samples were analyzed using FV3000 confocal microscopy.

### Migration assay

The Boyden chamber migration assay was conducted to evaluate cell migratory activity in A2058 cells using a gelatin-coated membrane with a pore size of 8 μm in diameter. A2058 cells with the expression of GFP (control), wild-type TM (TMG), or TM mutants (TM3A or TM-LeD) were loaded in the upper chamber with medium containing 0.1% FBS, and medium containing 0.5% FBS was applied in the bottom chamber as the chemoattractant. Cells were allowed to migrate for 3 h and then stained with Liu’s stain as reported previously [10]. Cells that migrated to the bottom surface were enumerated.

### Time-lapse recording and cell protrusion activity

A2058 cells expressing GFP, wild-type TM, or TM mutants were placed on Matrigel within an inverted microscope (Leica DMI6000 B) equipped with a cell culture system for time-lapse recordings. The relative cell protrusion activity was calculated by dividing the number of cell protrusions at the 1-h mark by the cell numbers at the time zero mark, as described previously [30].

### Mouse models

To study TM expression and the effect of soluble TMD1 on tumor growth in vivo, tumor xenograft and experimental lung metastasis models were conducted in NOD-SCID mice, as described in previous reports with substantial modifications [16, 31]. All mice were maintained in a specific pathogen-free animal facility at the Laboratory Animal Center at the authors’ university. Animal care conditions and the experimental protocols (109265 and 111329) were approved by the Institutional Animal Care and Use Committee at the authors’ university. For the tumor xenograft study, 7-week-old male NOD-SCID mice were subjected to subcutaneous injection of MeWo cell solution in PBS (1E6 cells/mouse) after systemic anesthesia and topical disinfection. The experimental mice were randomly assigned to the treatment. The rTMD1 (1 mg/kg) or PBS (vehicle control) was then given intraperitoneally once every 2 days. The change in subcutaneous tumor volume was monitored regularly with a digital caliper, and tumor weight was measured till the end of the experiment. To evaluate the effect of TMD1 on melanoma metastasis, an experimental lung metastasis mouse model was conducted in 7-week-old female NOD-SCID mice inoculated intravenously with MeWo cells (2E6 cells/mouse). The experimental mice were randomly assigned to receive either Dulbecco’s PBS (DPBS) as a vehicle control or AAV-TMD1 [16] once intravenously 4 days after tumor inoculation. Forty-three days after tumor injection, the animals were sacrificed, and the lung metastasis was evaluated.

### Immunohistochemistry

Paraffin-embedded tissue sections, 5 μm in thickness, were processed for rehydration. After rehydration, the antigen retrieval procedure was carried out by boiling the tissue section in sodium citrate, pH 6.0. Then, the tissue sections were incubated with the blocking solution, 1% bovine serum albumin in PBS, for 1 h at room temperature after 3.5% H_2_O_2_ treatment. The primary antibodies against human CD31, human melan-A, mouse CD31, or human TM were diluted in the blocking solution and applied to the tissue section for incubation at 4 °C for 1 day, followed by horseradish peroxidase-conjugated or alkaline phosphatase-conjugated secondary antibodies at room temperature for 2 h. The samples were washed properly with PBS supplemented with Tween-20 after each step of antibody incubation. The chromogen, including diaminobenzidine (Dako) and HIGHDEF^®^ Green AP Substrate (Enzo Life Sciences), was applied to the samples separately, followed by counterstaining with hematoxylin (ScyTek Laboratories). The Periodic Acid Schiff (PAS) stain was performed to detect the basement membrane following the experimental procedures (Baso Biotech CO., LTD). To differentiate between angiogenic and non-angiogenic tumor vasculature in human tissue samples and mouse tumor models, whole stained tissue sections were scanned using the TISSUEFAXS PLUS system, equipped with a 40 × oil-immersion objective lens, along with TissueQuest and HistoQuest image analysis software. Vessel-like structures positive for both CD31 and TM (CD31⁺/TM⁺) were classified as angiogenic vessels, whereas those positive for TM but negative for CD31 (CD31^−^/TM⁺) were classified as non-angiogenic vessels. The extent of tumor vasculature was semi-quantitatively scored as follows: −, 0; + , 1 ~ 10; +  + , 11 ~ 20; +  +  + , > 20, based on the number of corresponding vessels per tissue section.

### Statistical analysis

Data are expressed as mean ± standard error of the mean. Statistical analyses were performed with Student’s *t*-tests or one-way ANOVA followed by Bonferroni multiple comparison tests when more than two groups were compared. Values of *P* < 0.05 were considered statistically significant. Detailed information on statistical tests is provided in the figure legends.

## Results

### TM expression in angiogenic and non-angiogenic cancer vasculatures in melanoma

**Fig. 1:**
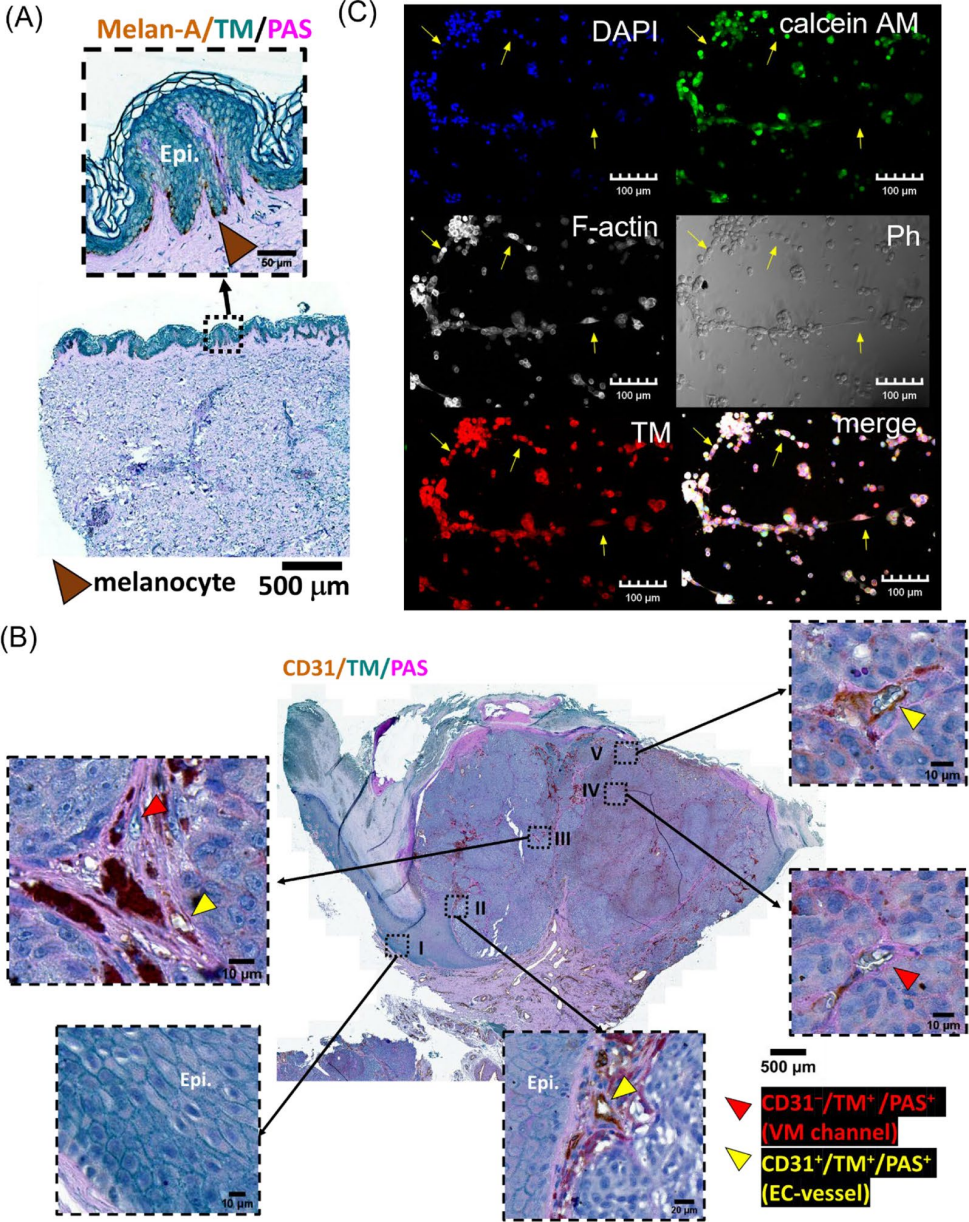
TM expression is associated with cancer angiogenic and non-angiogenic vasculatures. **A** Representative immunohistochemistry images of a normal abdominal skin biopsy displaying melan-A (brown), TM (green), and PAS (magenta). Epi. indicates the epidermis. **B** Representative immunohistochemistry images of a skin biopsy from the patient with cutaneous melanoma displaying CD31 (brown), TM (green), PAS (magenta), and nucleus (hematoxylin, deep blue-purple). The skin sample was derived from the right sole with a Clark’s level IV melanoma lesion. Angiogenesis is constructed by endothelial cells that display CD31^+^ and TM^+^ signatures (yellow arrows). Non-angiogenic vasculatures, referring to vascular mimicry (VM), are organized by cancer cells that display TM^+^ and CD31^−^ signatures (red arrows). **C** Representative confocal images of the co-culture of calcein AM-labeled MeWo cells (green) and HUVECs on Matrigel. Cells were stained for the nucleus (DAPI, blue), TM (red), and F-actin (white) on Matrigel at 4 h. The bright field image (Ph) and the merged channel (DAPI + calcein AM + TM + F-actin) present the incorporation of HUVECs (yellow arrows) in the MeWo VM network

The heterogeneous expression of TM in melanoma cell lines and surgical specimens has been noticed [25], while the functional correlation of TM expression in melanoma tumor progression remains unclear. In the current study, we analyzed the biological function of TM expression in melanoma tumor progression. Interestingly, the TM mRNA expression level is positively associated with patient survival in sarcoma, liver hepatocellular carcinoma, and kidney renal clear cell carcinoma, whereas inversely correlated with patient survival in cervical squamous cell carcinoma, esophageal squamous cell carcinoma, stomach adenocarcinoma, and lung squamous cell carcinoma (Table 1). In addition, a trend toward an inverse correlation was observed between overall survival in melanoma patients and the mRNA expression levels of TM (gene symbol THBD) and its associated molecules, including ezrin (EZR) and FAK (PTK2), as well as their combined expression, although these analyses did not reach statistical significance (Figure S1). These findings suggest that TM may exert distinct biological functions across different types of cancer. Analyzing TM expression in the normal cutaneous skin (Fig. 1A and Figure S2) and cutaneous melanoma (Fig. 1B, S3-S5) with the staining of endothelial marker CD31, basement membrane (PAS stain), or melanocyte/melanoma marker melan-A, we showed that TM was expressed in the epithelium (Fig. 1A and the inserts I and II in Fig. 1B), melanocyte (Fig. 1A), endothelium (Figure S4 and the inserts II, III, and V in Fig. 1B), and melanoma tumor (Fig. 1B and S4-S5) when compared to the control staining (Figure S2B and S3). Based on these observations, we found that TM was expressed both on CD31^+^ vessels (tumor angiogenesis) and CD31^−^ vessels (VM) in the human skin melanoma biopsy (inserts III and IV in Fig. 1B). This result, along with the previous study, suggests that TM may be involved in the formation of both tumor angiogenic vessels and non-angiogenic vessels. Although only small amounts of the skin tissue biopsies were included in the current study, the positive correlation of Clark’s level among tumor angiogenesis and VM of skin melanoma biopsy was suggested (Table 2). Using an in vitro cell model with the co-culture of human melanoma cells and endothelial cells on Matrigel, we showed that TM was expressed in both melanoma and endothelial cells during tube formation (Fig. 1C). Having endothelial TM contributes to angiogenesis [14, 19], and melanoma TM promotes cell invasion [20]. We thus investigated the role of TM in tumor non-angiogenic vessels, in particular, the melanoma VM.

**Table 1:** The correlation between TM mRNA expression level and patient overall survival among different tumors

Tumor type	*P* value	HR	Median overall survival	
			Low expression cohort (months)	High expression cohort (months)
Cervical squamous cell carcinoma (N = 304)	0.0041	1.99	45.73	21.2
Esophageal squamous cell carcinoma (N = 81)	0.002	4.9	45.37	16.13
Stomach adenocarcinoma (N = 375)	0.0056	1.58	56.2	21.1
Lung squamous cell carcinoma (N = 501)	0.032	1.35	65.1	38.47
Ovarian cancer (N = 374)	0.074	1.3	46	40.43
Bladder carcinoma (N = 405)	0.088	1.29	55.67	28.63
Sarcoma (N = 259)	0.0016	0.52	35.77	81.6
Liver hepatocellular carcinoma (N = 371)	0.0092	0.63	27.9	70.53
Kidney renal clear cell carcinoma (N = 530)	0.018	0.69	33.07	46.8
Esophageal adenocarcinoma (N = 80)	0.055	0.51	20.33	46.73
Uterine corpus endometrial carcinoma (N = 543)	0.08	0.66	59.77	103.73
Breast cancer (N = 1090)	0.23	0.82	131.37	130.87
Lung adenocarcinoma (N = 513)	0.37	0.88	48.47	50.03

The correlation between TM mRNA expression and the median overall survival was analyzed using pan-cancer RNA-seq data by a Kaplan–Meier survival plot. The hazard ratio (HR) with 95% confidence intervals and the log-rank* P* value are calculated

**Table 2:** The correlation of Clark’s level among angiogenic and non-angiogenic vessels of skin melanoma biopsy

No	lesion site (melan A^+^)	Clark’s level	TM^+^/CD31^+^ (EC-vessel)	TM^+^/CD31^−^ (VM channel)
01	Abdomen	Melanocytic nevus	** − **	** − **
02	Right sole	Melanocytic nevus	** − **	** − **
03	Left sole	Melanocytic nevus	** − **	** − **
04	Right heel	Melanocytic nevus	** − **	** − **
05	Left first toe web	In situ	** + **	** − **
06	Right cheek	In situ	** + **	** − **
07	Left breast	II	** + **	** − **
08	Right big toe	II	** + + **	** + **
09	Right sole	IV	** + + + **	** + **
10	Right thumb	IV	** + + + **	** + **
11	Left sole	IV	** + + + **	** + + **
12	Right sole	IV	** + + + **	** + + **
13	Right 3nd-toe	IV	** + + + **	** + + + **

The immunohistochemistry analysis of the expression level of the vascular endothelial cell vessel (EC-vessel, TM^+^/CD31^+^) and the VM channel (TM^+^/CD31^−^) in the melan-A^+^ area among skin biopsies.−, 0; + , 0 to 10; +  + , 10 to 20; +  +  + , over 20

### Augmentation of melanoma VM activity by TM expression

**Fig. 2:**
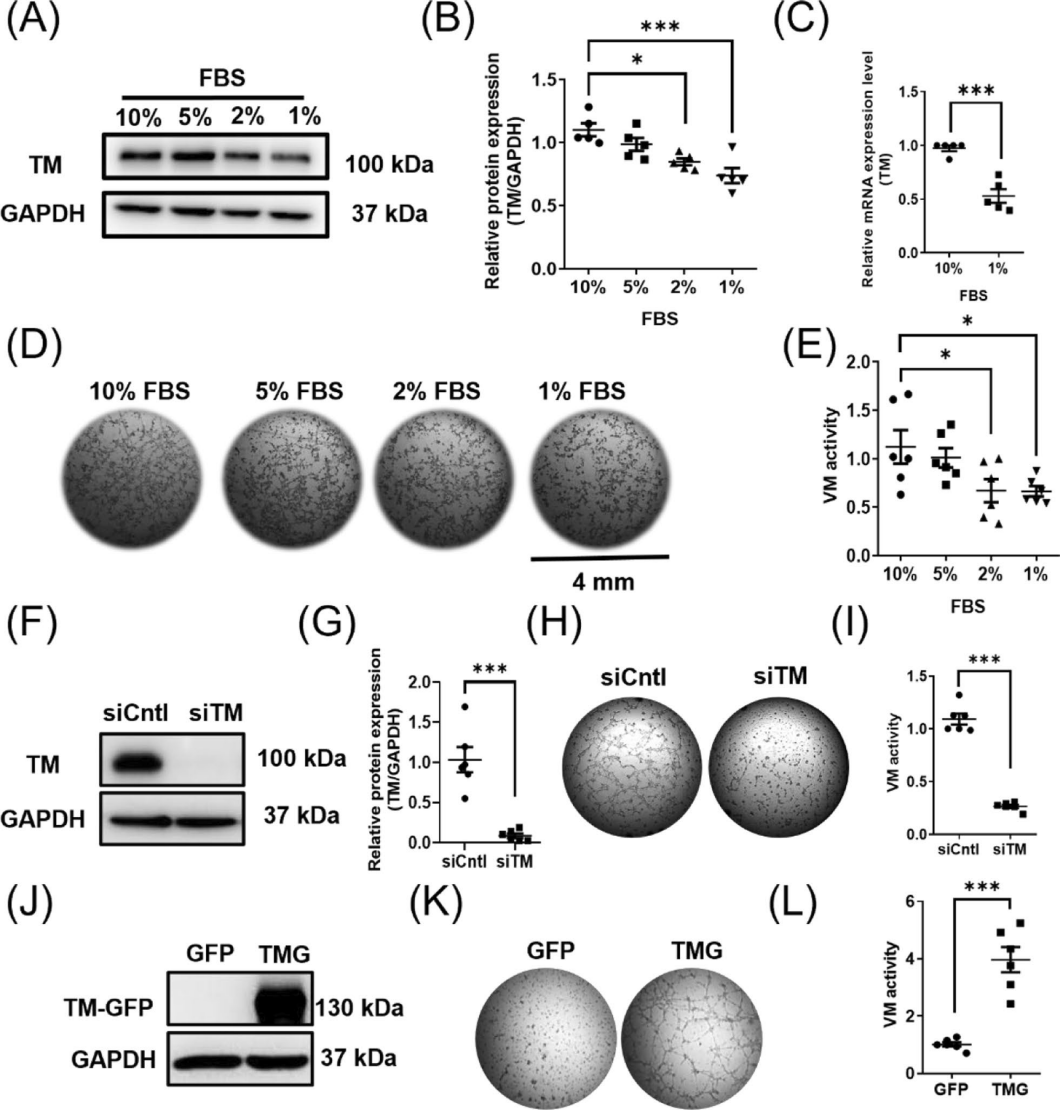
Augmentation of melanoma VM by TM expression. **A** Representative image of western blot of MeWo cells cultured with different serum concentrations for 1 day. Analysis of **B** TM protein expression level or **C** TM mRNA expression level in MeWo cells cultured with different serum concentrations for 1 day. N = 5. *P < 0.05; ***P < 0.01. **D** Representative images of tube formation (VM activity) in MeWo cells cultured with different serum concentrations for 1 day before being cultured on Matrigel for 2 h and **E** the statistical analysis thereof. N = 6. *P < 0.05. **F**–**I** Knockdown of TM using siRNA in MeWo cells suppressed VM activity on Matrigel. **F** Representative western blot of TM expression in MeWo cells with siRNA against TM (siTM) or control (siCntl), and **G** the statistical analysis thereof. N = 6. ***P < 0.001. **H** Representative images of tube formation in MeWo cells cultured on Matrigel for 5 h and **I** the statistical analysis thereof. N = 6. ***P < 0.001. (J-L) Overexpression of GFP-tagged TM (TMG) increased VM activity in the TM-null A2058 cells. **J** Representative western blot of TMG expression in A2058 cells. **K** Representative images of tube formation in A2058 cells cultured on Matrigel for 7.5 h and **L** the statistical analysis thereof. N = 6. ***P < 0.001

TM expression is regulated by several factors in different cell types [32]. Of note, TM is heterogeneously expressed in human malignant melanoma cells, partly due to TM promoter methylation [25]. In the current study, we found that the melanoma TM expression level was regulated by serum concentration (Fig. 2A). The reduction of serum concentration in the culture medium resulted in a decrease in TM protein expression level (Fig. 2B) and TM mRNA expression level (Fig. 2C), indicating that melanoma TM expression was, at least in part, transcriptionally regulated by unknown factors in the serum. Melanoma cells constructed a tube-like network on the Matrigel (VM activity) in a manner dependent on serum concentration (Fig. 2D). With higher serum concentration during cell culture before VM assay, a higher level of VM activity was observed (Fig. 2E), indicating that VM activity was boosted by serum and maybe by the induction of TM expression. Using gain- and loss-of-function strategies, we investigated the role of melanoma TM in tumor VM. TM expression was markedly suppressed by siRNA targeting TM in MeWo cells (Fig. 2F) to the extent of about 90% reduction (Fig. 2G). The VM activity was diminished in cells with TM-knockdown (Fig. 2H, I), mirroring the effects observed under low serum culture conditions. We thus used a gain-of-function strategy utilizing GFP-tagged TM (TMG) expression in TM-null melanoma cells (A2058 cells, A2058-TMG, Fig. 2J) to evaluate the function of TM in melanoma VM activity. The VM activity was notably low in TM-null melanoma cells (GFP-expressing A2058 cells, Fig. 2K), while significantly increased upon the introduction of TMG (Fig. 2L). These results indicated that TM expression augments melanoma VM activity.

### TM expression promotes melanoma cell-extracellular matrix (ECM) interactions

**Fig. 3:**
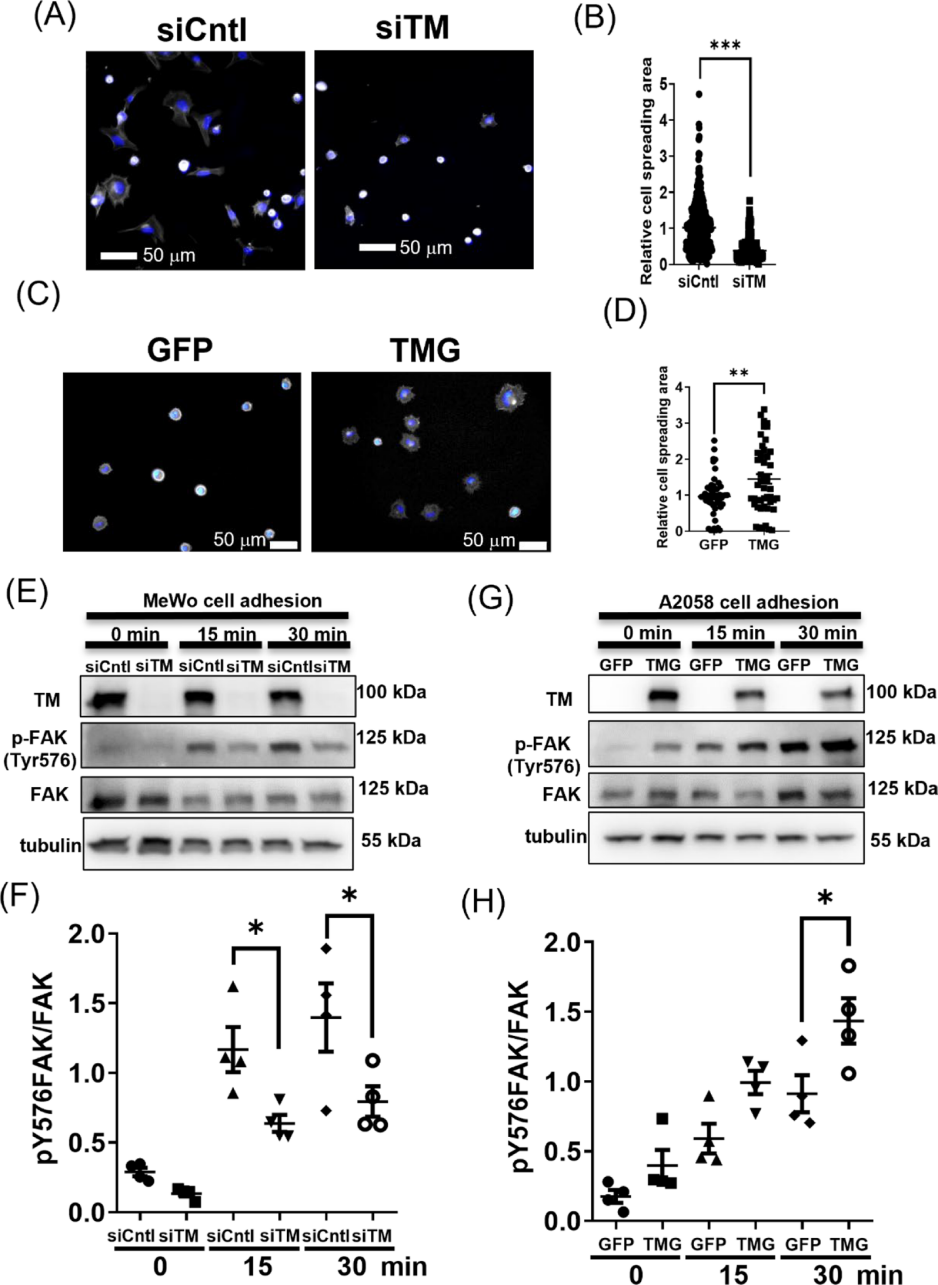
TM expression promotes melanoma cell-ECM interactions. Knockdown of TM in MeWo cells reduced cell spreading. **A** Representative images of the staining of microfilaments (white) and nucleus (blue) in MeWo cells with siRNA against TM (siTM) or scramble control (siCntl). The cells were allowed to adhere to the laminin-coated surface for 30 min. **B** Quantification of relative cell spreading area in **A**. N = 419 for siCntl and N = 290 for siTM. ***P < 0.001. (C and D) Overexpression of TM in A2058 cells promoted cell spreading on Matrigel. **C** Representative images of the staining of microfilaments (white) and nucleus (blue) in A2058 cells on a Matrigel-coated surface for 15 min. **D** Quantification of relative cell spreading area in **C**. N = 50 for both the GFP and TMG groups. **P < 0.01. **E** and **F** Western blot analysis of focal adhesion activity in MeWo cells. **E** Representative images of western blot of MeWo cells with siCntl or siTM onto Matrigel-coated surface for 0, 15, and 30 min, and **F** the statistical analysis thereof. N = 4. *P < 0.05. (G and H) Western blot analysis of focal adhesion activity in A2058 cells. **G** Representative images of western blot of A2058 cells with GFP or TMG onto Matrigel-coated surface for 0, 15, and 30 min, and **H** the statistical analysis thereof. The ratio of p-FAK Tyr576 over FAK among the groups was analyzed. N = 4. *P < 0.05

Given that cell-ECM adhesion, remodeling, and migration activity are crucial for melanoma VM activity [10, 33], we investigated the role of TM expression in melanoma cell-ECM interaction by analyzing cell spreading activity and focal adhesion signaling. MeWo cells spread out on laminin as illustrated by microfilament staining (Fig. 3A). The cell spreading area was reduced in MeWo cells with TM-knockdown (Fig. 3A, B). A consistent result was observed in the TM gain-of-function system. A2058 cells were insufficient in cell spreading on Matrigel in short periods of adhesion (Fig. 3C). When TM was overexpressed in A2058 cells, the cell spreading activity was increased (Fig. 3C, D). These results from cell spreading activity among TM-expressing and TM-null melanoma cells implied that TM may modulate cell-ECM interaction or focal adhesions. It is known that multiple steps of tyrosine phosphorylation are involved in the FAK activation cascade [34]. Among the phosphorylation sites, the prime state (pY397) and fully active state (pY576) of FAK have been associated with melanoma VM activity [35]. Notably, TM expression influences these two activation steps of FAK during tumor angiogenesis, as demonstrated in our previous study [19]. Therefore, we analyzed focal adhesion signaling relevant to VM condition specifically in terms of the phosphorylation level of pY576FAK. The results showed that the activating phosphorylation level of FAK (pY576-FAK) was associated with TM expression level during cell-ECM interaction. The level of FAK activation was reduced in the TM-knockdown MeWo cells (Fig. 3E, F) while increased in the A2058-TMG cells in a time-dependent manner (Fig. 3G, H). The expression of the VM-relevant membrane molecule CD133 in melanoma cells [36] was not changed with TM alteration (Figure S6). These results implied that TM may be involved in melanoma cell-ECM interaction.

### Suppression of focal adhesion impedes TM-mediated cell migration and VM activity in melanoma cells

**Fig. 4:**
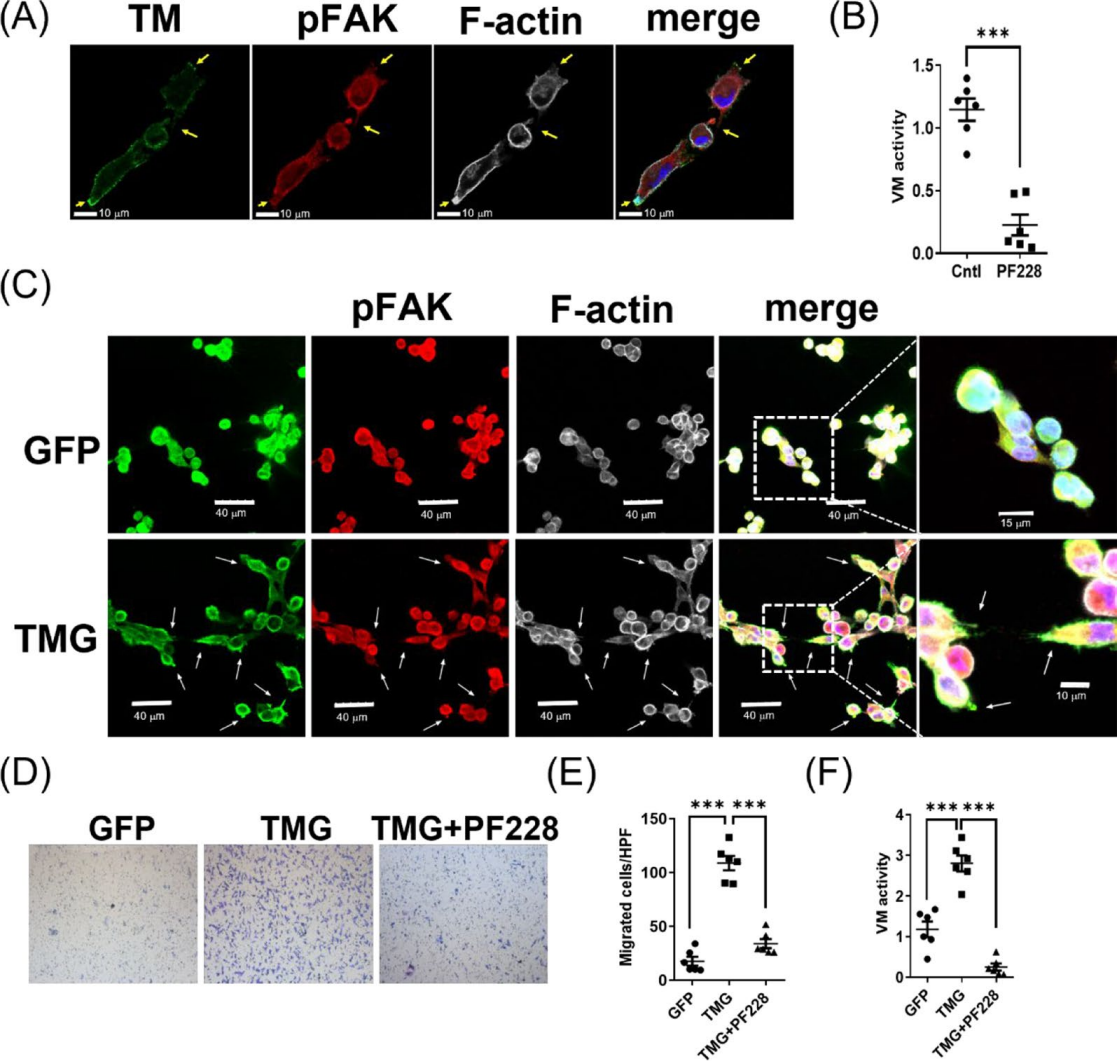
Suppression of focal adhesion impedes TM-mediated cell migration and VM activity in melanoma cells. **A** Representative confocal images of TM and pY576FAK staining in MeWo cells at the early stage (1 h) of tube formation on Matrigel. **B** The inhibition of VM activity in MeWo cells with PF228 treatment. N = 6. ***P < 0.001. **C** Representative confocal microscopy images of pY397FAK and F-actin staining in A2058-GFP or A2058-TMG cells at the early stage (1 h) of tube formation on Matrigel. **D** Representative images of Boyden chamber migration assay. The GFP- or GFP-tagged TM (TMG)-expressing A2058 cells with or without FAK inhibitor (PF-228, 10 mM) were loaded into the upper compartment of the Boyden chamber with a gelatin-coated membrane and allowed to migrate in response to 0.5% FBS. After 3 h, migrated cells on the filter's lower surface were stained with Liu’s stain. **E** Statistical analysis of migration assay. N = 6. ***P < 0.001. **F** Inhibition of FAK by treatment with PF-228 suppressed VM activity in A2058 cells. N = 6. ***P < 0.001

The focal adhesion signaling is critical for cell adhesion and migration. In particular, FAK regulates cell-ECM interaction and VM [37–39]. Of note, endothelial TM plays an important role in FAK activation, tube formation, and tumor angiogenesis [19]. We thus investigated the distribution of TM and FAK in melanoma cells that underwent VM progression. At the early stage of VM, we observed a close association of TM and the activated FAK at cell protrusions in MeWo cells (Fig. 4A), suggesting that TM and FAK may participate in cell migration and VM activity. Indeed, suppression of FAK activation using FAK inhibitor, PF228, significantly inhibited VM activity in MeWo cells (Fig. 4B) as observed in TM-knockdown cells (Fig. 2F–I). Thus, we further investigated the role of FAK in TM-mediated melanoma cell migration and VM using A2058 cells. Compared with GFP-expressing A2058 cells, activated FAK was colocalized with TM and F-actin at cell protrusions in TMG-expressing A2058 cells before tube connection on Matrigel (Fig. 4C). Additionally, we found that TM-mediated melanoma cell chemotactic migration was dependent on FAK activation (Fig. 4D). Melanoma cell migration was enhanced upon TM expression, while subsiding to nearly basal levels in cells treated with PF228 (Fig. 4E), indicating that FAK may be downstream of TM in mediating melanoma cell adhesion and migration. These results implied that FAK may contribute to melanoma VM activity. Therefore, we further analyzed whether FAK participates in TM-mediated VM in melanoma cells. Melanoma VM activity was enhanced by TM expression, whereas it was diminished when FAK activation was suppressed (Fig. 4F). These results indicated that focal adhesion signaling, particularly FAK activity, could be downstream of TM expression in mediating melanoma cell adhesion, migration, and VM activity.

### Ezrin contributes to TM-mediated melanoma cell migration and VM activity

**Fig. 5:**
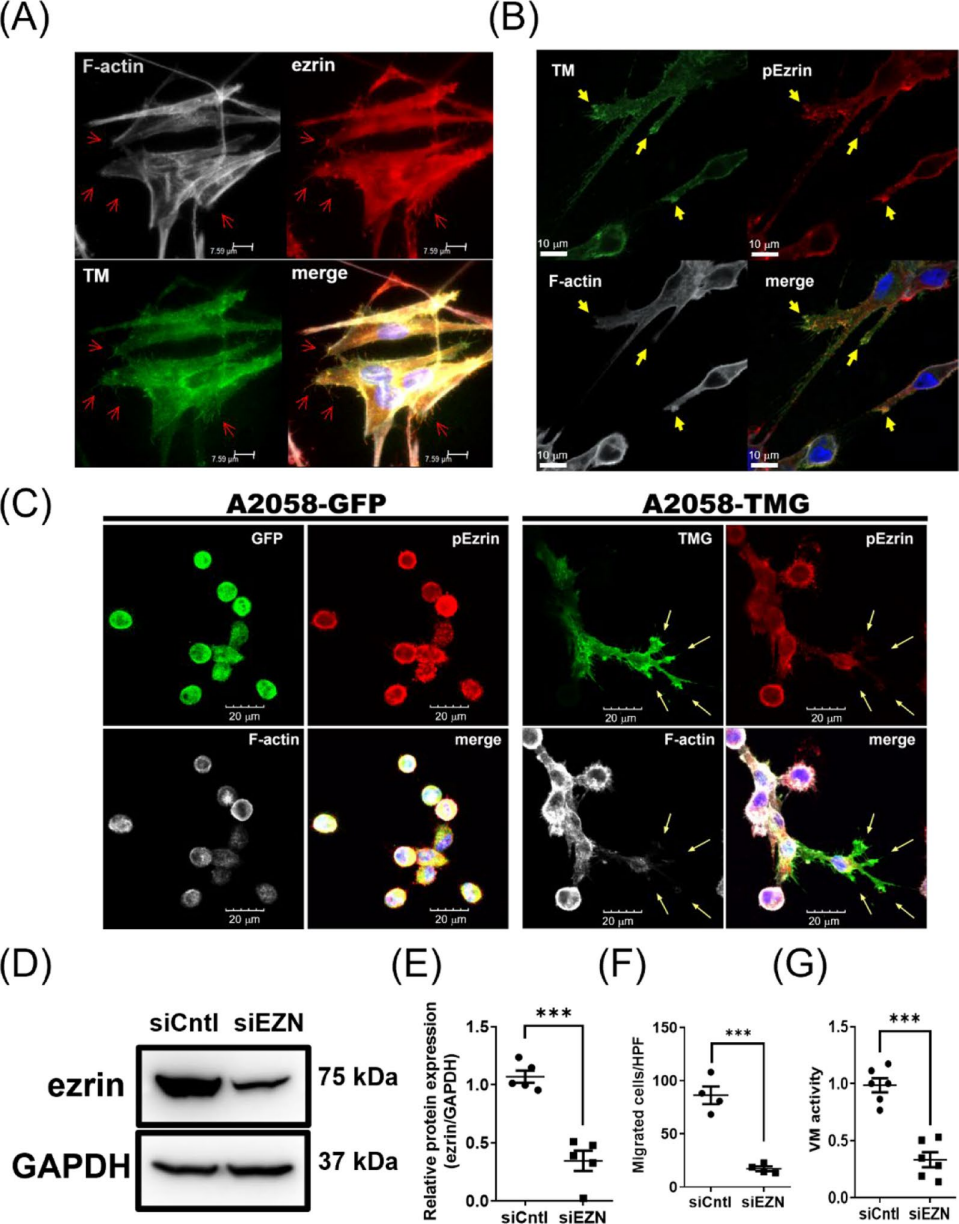
Ezrin contributes to TM-mediated melanoma cell migration and VM. **A** Representative widefield images of MeWo cells stained for nucleus (DAPI, blue), TM (green), F-actin (white), and ezrin (red) on the laminin-coated surface. Red arrows indicate filopodia. **B** Representative confocal images of MeWo cells stained for the nucleus (DAPI, blue), TM (green), F-actin (white), and ezrin (red) on Matrigel at 1 h. Arrows indicate cell protrusions and filopodia. **C** Representative confocal images of GFP- and TMG-expressing A2058 cells on Matrigel at 1 h. The cells were stained for the nucleus (DAPI, blue), pEZN (red), and F-actin (white). Arrows indicate cell protrusions. (D-G) Knockdown of ezrin abolished TM-mediated cell migration and VM activity in A2058 cells. **D** Representative western blot of TMG-expressing A2058 cells with siRNA targeting ezrin (siEZN) or control (siCntl) and **E** the statistical analysis thereof. N = 5. ***P < 0.001. The cells were subjected to **F** Boyden chamber migration assay (N = 4) and **G** VM activity assay on Matrigel (N = 6). ***P < 0.001

Cytoskeleton remodeling plays an important role in cell migration and morphogenesis. Actin-binding protein, ezrin, regulates FAK activation [40], cytoskeletal reorganization, filopodia formation, and cancer cell invasion [41, 42]. In particular, filopodia on tip endothelial cells and tumor cells lead the way for vessel branching and collective invasion, respectively [43, 44]. In tip endothelial cells, TM expression and its association with ezrin are critical for cell migration during sprouting angiogenesis [14]. Therefore, we wondered whether ezrin plays a role in TM-mediated melanoma cell migration and VM. The results showed that TM and ezrin were associated with microfilaments at filopodia in MeWo cells under 2D culture conditions (Fig. 5A). Whether this association remains important during the VM process on Matrigel was further investigated. As a result, cells became polarized on Matrigel before cell–cell connections, and the functional ezrin, the phosphorylation on Thr567 of ezrin that links the microfilaments, was colocalized with TM at cellular protrusions and filopodia (Fig. 5B), suggesting that ezrin may be involved in TM-mediated cell migration and VM activity. Though the expression and the phosphorylation level of ezrin were not changed by TM expression (Figure S7), the subcellular distribution of the functional ezrin during the VM process was associated with TM expression (Fig. 5C). The functional ezrin was mainly localized around the cell membrane, both in GFP- and TMG-expressing A2058 cells. However, TM expression causes cell polarization with ezrin localization at cell protrusions (arrows in Fig. 5C), suggesting that the interaction of TM and ezrin may be responsible for cell migration and VM activity. Therefore, we further investigated cell migration and VM in the context of ezrin knockdown in the A2058-TMG cells. The expression of ezrin was successfully reduced by siRNA targeting ezrin in A2058-TMG cells (Fig. 5D) to the extent of about 70% reduction (Fig. 5E). Knockdown of ezrin did not alter FAK activation (Figure S8). Still, it abolished TM-mediated cell migration (Fig. 5F) and VM activity (Fig. 5G), indicating that ezrin is critical for melanoma migration and the VM process enhanced by TM expression. These results suggest that TM promotes melanoma VM activity, likely through two separate pathways—ezrin and FAK.

### The lectin domain and the ezrin-binding motif of TM are required for TM-mediated melanoma cell migration and VM

**Fig. 6:**
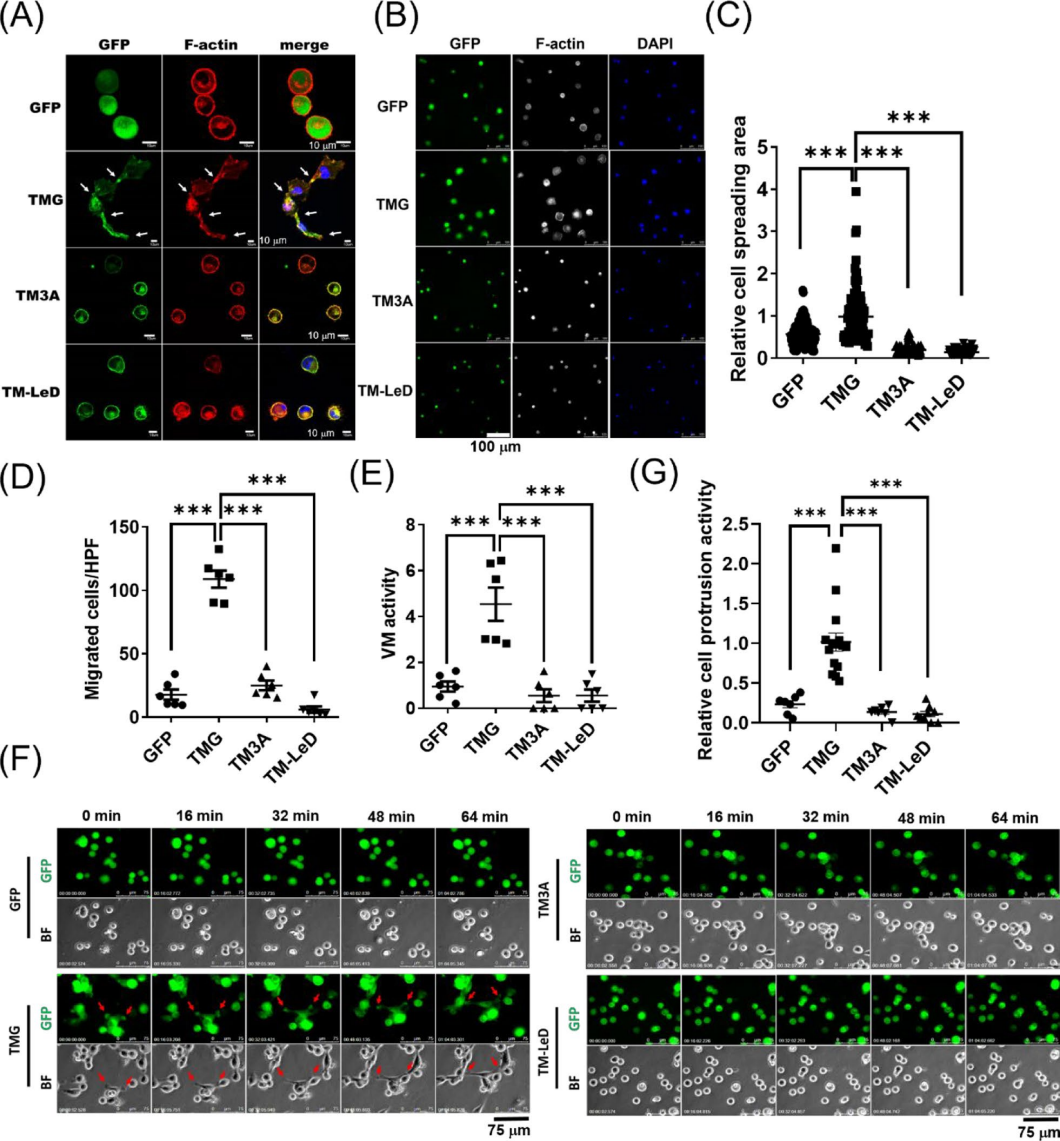
The lectin domain and the ezrin-binding motif of TM are required for TM-mediated cell migration and VM in melanoma cells. A2058 cells were transfected with various GFP-tagged TM-expressing vectors, including wild-type TM (TMG), mutations at the TM cytoplasmic domain (^522^RKK^524^ to ^522^AAA^524^_,_ TM3A), and lectin-like domain-deleted TM (TM-LeD). **A** Representative confocal images of GFP and F-actin in A2058 cells with different TM mutants on Matrigel at 1 h. TM co-localized with F-actin at cell protrusions (white arrows). **B** Representative images of the staining of microfilaments (white) and nucleus (blue) in A2058 cells on a Matrigel-coated surface for 30 min, and **C** the quantification of relative cell spreading area thereof. N = 100 for each group. ***P < 0.001. A2058 cells were subjected to **D** Boyden chamber migration assay with serum gradient for 3 h and **E** tube formation assay on Matrigel for 3 h. N = 6. ***P < 0.001. **F** Representative images of cell–cell associations during tube formation on Matrigel in A2058 cells. The images were taken from the time-lapse recordings of A2058 cells on Matrigel. The bottom left stamp shows the relative period (h:min:sec:ms). BF indicates the bright-field channel. GFP indicates the GFP channel. Red arrows indicate cell–cell associations. **G** Quantification of cell protrusion activity at the early stage of tube formation per high power field (HPF) in **F**. N = 7 HPF for the GFP group, which included 459 cells. N = 15 HPF for the TMG group, which included 920 cells. N = 7 HPF for the TM3A group, which included 193 cells. N = 9 HPF for the TM-LeD group, which included 454 cells. ***P < 0.001. The cell protrusion activity was calculated by dividing cell protrusion numbers at the 1-h mark by the cell numbers at the time zero mark

The multiple domains of TM endow diverse biological functions in different cell types [45]. For example, its lectin domain and ezrin-binding motif are necessary for endothelial podosome formation, migration, and tube formation [14]. We thus investigated TM functional domains in melanoma cell adhesions, migration, and VM processes using TM mutants, including lectin-deleted TM (TM-LeD) and ezrin-binding motif-mutated one (TM3A), with GFP (vector control) as the negative control and TMG as the positive control in A2058 cells. When cells were cultured on Matrigel, except for TMG cells, none of the TM mutants or vector control cells showed protrusive phenotypes. TM was polarized at F-actin-enriched cell protrusions in TMG cells before VM network formation (Fig. 6A). Compared with TMG, cell-ECM adhesion activity, as measured by cell spreading area on Matrigel-coated surface, was reduced in TM mutants (Fig. 6B, C). These results indicated that both lectin and ezrin-binding motifs were required for TM-mediated cell-ECM interaction and cell protrusions. In addition, not only chemotactic cell migration (Fig. 6D) but also VM activity (Fig. 6E and videos 1 and 2) of melanoma cells enhanced by TM expression were abolished when its lectin domain or ezrin-binding motif was mutated, highlighting the importance of these two functional domains in mediating aggressive phenotypes. In agreement with these results, TM was found to be polarized at cell protrusions and actively involved in mediating cell–cell association before tube formation (arrows in Fig. 6F and videos 1 and 2). TM-mediated cell protrusion activity was significantly diminished in cells with TM mutants during the VM process (Fig. 6G). Altogether, our in vitro model of melanoma VM involving TM’s functional domains in mediating cell-ECM adhesion, cell–cell association, migration, and tube formation underscored the importance of its lectin domain and cytoplasmic domain in modulating aggressive phenotypes and strongly suggested that targeting TM’s functional domains for tumor suppression.

### Soluble TMD1 inhibits melanoma VM activity and melanoma tumor progression in vivo

**Fig. 7:**
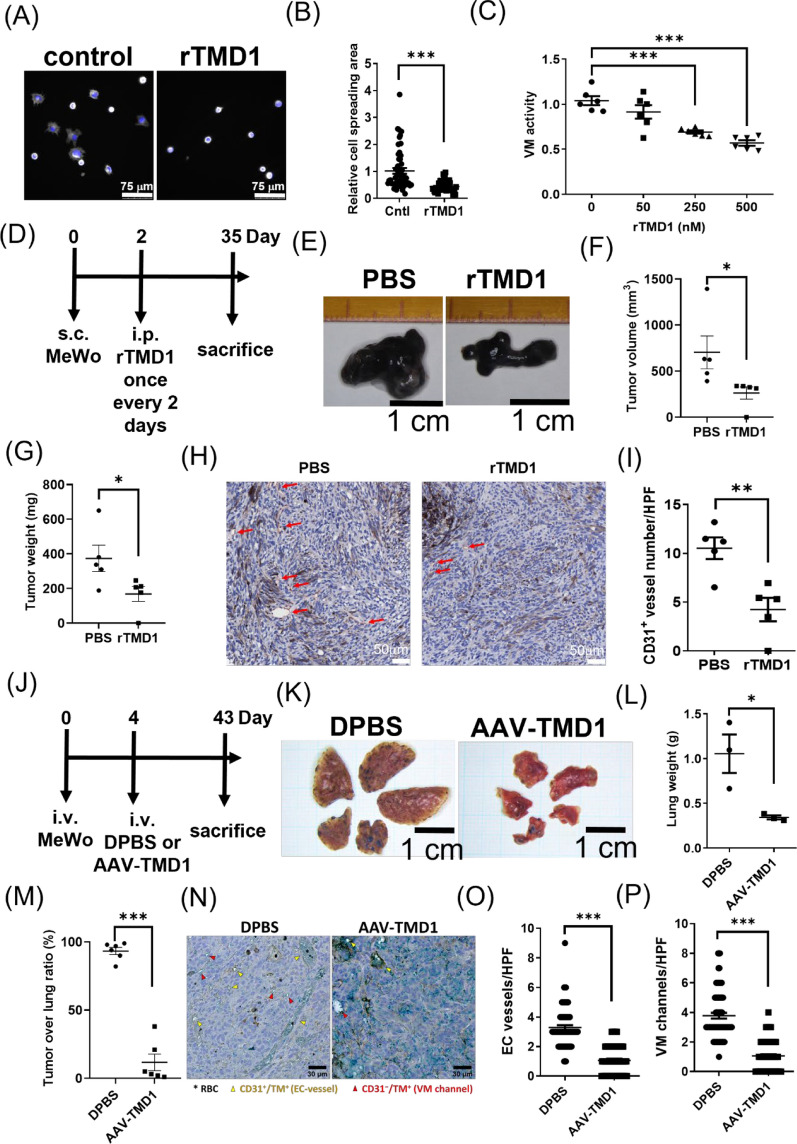
Soluble TMD1 inhibits melanoma VM activity and melanoma tumor progression in vivo. **A** and **B** rTMD1 interfered with MeWo cell spreading on Matrigel. **A** Representative images of MeWo cells spreading on Matrigel at 30 min with rTMD1 (500 nM). **B** Statistical analysis of the relative cell spreading area in **A**. N = 52. ***P < 0.001. **C** rTMD1 reduced VM activity in MeWo cells. N = 6. ***P < 0.001. **D**–**I** rTMD1 inhibited melanoma tumor growth and tumor angiogenesis in vivo in a MeWo cell xenograft model. **D** Experimental protocol of tumor xenograft. s.c., subcutaneous injection. i.p., intraperitoneal injection. **E** Representative images of the melanoma tumor xenograft at day 35. Statistical analysis of **F** tumor size and **G** tumor weight at day 35. N = 5. *P < 0.05. **H** Representative immunohistochemistry staining of CD31 (brown) in the tumor sections. Red arrows indicate CD31^+^ endothelial vasculature. **I** Statistical analysis of CD31^+^ vessels per high power field (HPF). N = 5. **P < 0.01. **J**–**P** AAV-TMD1 inhibited melanoma lung metastasis and tumor vasculatures, including the endothelial vessels and VM channels in vivo. **J** Experimental protocol of lung metastasis. i.v., intravenous injection. **K** Representative images of melanoma lung metastases. **L** Statistical analysis of the total lung lobes' weight per mouse. N = 3. *P < 0.05. **M** Statistical analysis of the tumor occupancy in the lung section. Two lung lobes from each mouse were analyzed. N = 6. ***P < 0.001. **N** Representative images of CD31 (brown) and TM (green) stains on the lung section. The yellow arrowheads indicate endothelial cell (EC) vessels (CD31^+^ and TM^+^), and the red arrowheads indicate VM channels (CD31^−^ and TM^+^). Statistical analysis of **O** EC vessels and **P** VM channel per high power field (HPF, 40X) in the lung tumor nodules. N = 73 from 6 lung lobes in each group. ***P < 0.001

Both tumor angiogenesis and VM facilitate malignant tumor progression. Therefore, the therapeutic strategy inhibiting tumor angiogenesis and VM simultaneously would be beneficial in suppressing tumor growth and dissemination. VEGF pathway plays a dominant role in tumor angiogenesis and regulates tip endothelial cell phenotypes, in part, through upregulation of TM [14, 19]. We thus investigated the VEGF pathway in melanoma VM using VEGF treatment in combination with anti-VEGF. Indeed, VEGF stimulated endothelial tube formation on Matrigel, which was suppressed by the addition of anti-VEGF antibody (Figure S9A). Nonetheless, VEGF or in combination with anti-VEGF antibody had no significant effect on melanoma VM activity (Figure S9B), suggesting that the VEGF pathway may affect endothelial tube formation but not melanoma VM. Given that our in vitro study has indicated the essential role of the TM lectin-like domain in melanoma cell adhesion, migration, and VM, we contemplated intervening in tumor progression by targeting TM's lectin-like domain using the decoy molecule, soluble TMD1. Though the melanoma cell proliferation was not affected by rTMD1 treatment within 3 days of culture in vitro (Figure S10), melanoma cell adhesion activity on Matrigel was significantly reduced by rTMD1 treatment (Fig. 7A, B). Moreover, rTMD1 suppressed melanoma VM activity in a concentration-dependent manner (Fig. 7C). These results imply that rTMD1 may possess anti-tumor activity. In line with these, melanoma tumor growth in vivo (Fig. 7D) was significantly suppressed by rTMD1 treatment (Fig. 7E). Tumor mass in mice treated with PBS (vehicle) showed greater growth (Fig. 7F) and increased weight (Fig. 7G) compared to those in mice receiving rTMD1 treatment, indicating the efficacy of rTMD1 in mitigating tumor growth. We thus investigated tumor microvessel density. The CD31-positive vessels were revealed throughout the tumor sections in both PBS- and rTMD1-treated groups (Fig. 7H). Tumor angiogenesis was reduced in the rTMD1-treated group when counting the CD31-positive vessels among the xenograft tumor tissue sections (Fig. 7I). Though the VM activity was difficult to determine in the MeWo cell xenograft model using the PAS staining method, our results showed anti-tumor activity of rTMD1 in vitro and in vivo. The therapeutic potential of soluble TMD1 on melanoma lung metastasis was further evaluated using an adeno-associated virus carrying TMD1 transgene expression (AAV-TMD1) that enables sustained generation of soluble TMD1 in the circulation by host hepatocytes [16]. In this model, MeWo cells were intravenously injected into the experimental mice 4 days before AAV-TMD1 treatment (Fig. 7J). Metastatic tumor nodules were observed on the lung surface in all experimental groups. In contrast to the control group (treated with the vehicle, DPBS), mice administered AAV-TMD1 exhibited a notable reduction in metastatic foci and lung size (Fig. 7K). Moreover, a significant increase in lung weight was observed in DPBS-treated mice compared to those treated with AAV-TMD1 (Fig. 7L), suggesting a significant growth of tumors on the surface and within the lung tissues. Indeed, immunohistochemistry analysis of TM and CD31 expression in the lung section (Figure S11) revealed the loss of normal lung structure and profound growth of tumor loci in the control mice compared with AAV-TMD1-treated mice (Fig. 7M). Additionally, when analyzing the intratumoral vasculatures (Fig. 7N), we found that both tumor-angiogenesis (Fig. 7O) and VM (Fig. 7P) were reduced by AAV-TMD1 treatment. These findings contribute to our knowledge of melanoma TM in mediating tumor progression, and soluble TMD1 exhibited anti-tumor effects both in vitro and in vivo.

## Discussion

Despite 30 years of research warning that TM expression in cancer cells may modulate cancer cell behaviors and impact patient survival [21, 22], the biological function of cancerous TM and the underpinning molecular mechanisms remain elusive. Heterogeneous expression of TM among tumor cells and distinct correlations between TM expression and patient survival among different cancers (Table 1 and Figure S1) [23–25, 27] imply a controversial scenario of TM in tumor biology. This could be the pleiotropic nature of oncofetal antigens such as TM in various cell types and the result of the responses to different microenvironmental stimulations [45]. Our immunohistochemistry analysis of vasculature from thirteen human samples, ranging from normal skin to advanced melanoma tissues (Table 2), suggests a possible association between increased tumor vasculature, encompassing both angiogenesis and vascular mimicry, and Clark’s level. Nevertheless, the limited sample size may constrain the generalizability of these findings. Our current study reveals TM's pro-migratory and pro-VM function in aggressive melanoma cells through ezrin- and FAK-dependent mechanisms and demonstrates a potential therapeutic strategy against melanoma progression using TM decoy.

The diverse cellular functions of TM under various biological conditions have been noticed. TM expression in epithelial cells is correlated with the reduction of cell proliferation rate, in part, through promoting cell–cell junction formation and epithelial differentiation [12]. Whereas, epithelial TM promotes collective cell migration upon wound-induced regeneration [13]. In endothelial cells, TM expression is critical for maintaining a quiescent endothelium. Knockout of TM in vascular endothelial cells drives a pro-inflammatory phenotype [46]. Once activated by VEGF, endothelial TM plays an important role in organizing 3D podosomes that initiate sprouting angiogenesis [14]. Under pathogenic conditions, TM-mediated anti-coagulation involves pathogenic progression of cerebral cavernous malformations [47] and determines tumor metastasis [48]. In addition to endothelial TM, diverse biological functions of TM expression in cancer cells have been suggested. TM promotes cell–cell junction formation and slows down tumor growth independent of its anticoagulant activity [12, 27]. Once cells are activated and cell–cell junctions are dissociated, the TM expression in cancer cells enhances cell migration and invasion [19]. Notably, tube-like connections between tumor spheroids were observed exclusively in TM-expressing melanoma spheroids embedded within a 3D collagen gel [20], suggesting that TM may facilitate VM-like behavior under 3D conditions. Our study adds to an increasing body of evidence that the pleiotropic function of TM also applies to melanoma cell plasticity. It seems likely that tumor cell proliferation can be reduced when TM expression level is boosted to an extremely high level in TM-null cells, such as A2058 cells, through tightening the cell–cell junction formation [12]. This phenomenon may contribute to cancer dormancy in vivo. The current study showed that A2058 cells with TM expression became more aggressive in terms of cell migratory activity and VM activity upon cell dissociation in vitro. Reduction of TM expression in the aggressive melanoma cells (MeWo cells) resulted in diminished cell adhesion and VM activity. These results underscore the cellular context-dependent roles of TM in cell activities, which may reflect the onco-developmental processes. In addition, heterogeneity of TM gene expression levels in tumor cell populations in the tumor mass may contribute to the complexity of these seemingly contradictory observations.

VM phenotype is first described in human uveal melanoma tissue samples and then identified in different cancer types [8, 49]. Aggressive cancer cells assume VM capability to organize vessel-like structures through several mechanisms. Several markers for VM have been proposed. In particular, tumor cells' expression of endothelial-specific cell adhesion molecules VE-cadherin turns on the aggressive phenotypes with increased cell migration and VM activity [50]. Moreover, CD133^+^ melanoma subpopulations promote tumorigenicity through VM activity [51]. Additionally, the expression of stromal factors, such as CD248, regulating cell-ECM interactions in melanoma cells is critical for VM activity [10]. It seems interesting to notice that molecules involved in cell–cell or cell-ECM interactions and with stem-like activity have the potential to promote cell migration and VM activity. Of note, TM is rich in endothelial cells and keratinocytes, where it participates in cell–cell contact and cell migration. TM is also found to be expressed by fibroblasts, while its function is yet to be defined. Whether members of the C-type lectin family XIV proteins share similar biological functions in tumor VM should be of interest and warrant study.

In this study, we found that both TM expression and FAK activity are critical for VM activity. TM protein expression influenced VM activity (Fig. 2), FAK activity (Fig. 3), and its subcellular localization (Fig. 4). Moreover, inhibition of FAK in a TM gain-of-function system reversed TM-enhanced melanoma migration and VM activity, supporting the conclusion that FAK functions downstream of TM (Fig. 4). A similar relationship was observed with ezrin. TM expression in A2058 cells promoted ezrin localization at cell protrusions (Fig. 5C), whereas ezrin knockdown abrogated TM-induced increases in melanoma cell migration (Fig. 5F) and VM activity (Fig. 5G). Furthermore, mutation of the TM cytoplasmic domain responsible for ezrin binding eliminated TM-driven cell spreading, migration, protrusion, and VM activity (Fig. 6). Collectively, these findings suggest that both FAK and ezrin act as potential effectors downstream of TM. However, the causal relationship remains unresolved, as it is unclear whether TM expression itself is regulated by FAK or ezrin.

FAK has broad-spectrum biological functions and has been implicated as an emerging target for malignant cancers [34]. Studies have positioned FAK not merely as a kinase, but as a “plasticity switch” that drives VM in cutaneous and uveal melanoma through downstream signaling, involving the phosphorylation of FAK at Y397 and Y576 residues [35]. pY397 enables Src recruitment and downstream PI3K/Akt signaling, while pY576 (within the kinase activation loop) ensures maximal FAK catalytic activity [34]. Together, these phosphorylation events promote cytoskeletal remodeling, adhesion turnover, and extracellular matrix reorganization—key processes that allow melanoma cells to form vessel-like networks independent of endothelial cells. Consistent with these findings, TM expression facilitates cell adhesion, thereby enhancing VM activity through the FAK pathway. Although the mechanism by which TM expression promotes FAK activation remains unclear, and it is not yet known whether suppression of FAK signaling directly influences TM expression or the expression and activation of ezrin, the observed trend of the inverse correlation between overall patient survival and the expression levels of TM, ezrin, and FAK in cutaneous melanoma highlights the TM-ezrin and TM-FAK pathways as promising therapeutic targets for limiting melanoma progression.

In addition to pathological cancer progression, VM is also observed under the physiological condition that occurs during hemochorial placentation, where embryo-derived invasive trophoblasts participate in the remodeling of the uterine spiral arteries [52, 53]. The trophoblasts undergo a stepwise epithelial-to-endothelial transformation by which endothelial adhesion receptors are increased along the invasive path [53]. The transcriptome analyses of trophoblast subsets identify expression changes in genes associated with migration, cytokines, growth factors, and angiogenic factors [54]. Trophoblasts, along with endothelial cells, construct hemochorial maternal vascular spaces in the placenta through VM [55]. Of note, TM is expressed in trophoblast cells [56] and is essential for embryogenesis [57]. However, the pro-migratory and pro-cell adhesion features of TM in trophoblast VM progression are yet to be investigated.

Targeting endothelial cells as a strategy to wipe out the blood supply to tumors has been utilized for cancer therapy with moderate initial responses, while developing resistance in the long-term treatment. Several factors, including intrinsic factors that transform tumors or extrinsic factors affecting tumor microenvironments, may contribute to the resistance in anti-angiogenesis therapy [2, 58]. Of note, the increase of cancer cell plasticity via the transformation of tumors acquiring VM activity has been considered an important reason for resistance to anti-angiogenic therapy [4, 36]. Therefore, the combination of anti-angiogenesis and anti-VM therapies exhibits high potential to suppress tumor progression. In line with this, the recombinant TM lectin domain has been demonstrated to diminish breast cancer cell adhesion, suggesting its potential to interfere with breast cancer progression [59]. In this study, we identified TM expression in both tumor angiogenic and non-angiogenic vessels, suggesting that blocking TM functions might be able to suppress angiogenesis and VM. Indeed, we demonstrated that soluble TMD1, such as rTMD1 protein or AAV-TMD1, exhibited a suppression effect on human melanoma cells in both the mouse models of melanoma tumor xenograft and lung metastasis, in part, through anti-tumor angiogenesis and anti-VM, highlighting a substantial rationale for the application of soluble TMD1 as an anti-tumor reagent to subside tumor progression. While our findings provide substantial evidence that TM contributes to melanoma VM activity and that soluble TM may act as a decoy molecule, the alternative possibility that soluble TM exerts inhibitory effects through interactions with unidentified glycans cannot be excluded, given the broad glycan-binding capacity of lectin-like molecules.

## Conclusion

In summary, our findings provide strong evidence that TM expression in melanoma is correlated with tumor cell VM activity, implicating both ezrin and FAK in this process. Importantly, we report for the first time the presence of TM in both angiogenic and non-angiogenic vessels of skin melanoma, underscoring its potential role in tumor progression. Given TM's critical role in both angiogenesis and VM activity, it may serve as a novel target for antitumor therapies. As demonstrated in this study, soluble TMD1 can function as a decoy molecule, interfering with tumor progression in vitro and in vivo. We, therefore, conclude that melanoma TM promotes aggressive phenotypes, and soluble TMD1 can inhibit the progression of both angiogenic and non-angiogenic vessels in melanoma tumors.

## Supplementary Information

Supplementary material 1.

Supplementary material 2.

Supplementary material 3.

## Data Availability

Data and materials associated with this study will be available upon reasonable request from the corresponding author.

## Abbreviations

VM: Vascular mimicry
TM: Thrombomodulin
rTMD1: Recombinant TM lectin-like domain
FAK: Focal adhesion kinase
FBS: Fetal bovine serum
TM-LeD: Lectin-like domain-deleted TM
TM-^522^RKK^524^/AAA, TM3A: Ezrin-binding motif mutation of TM cytoplasmic domain
PBS: Phosphate-buffered saline
TMG: GFP-tagged TM
ECM: Extracellular matrix
AAV-TMD1: Adeno-associated virus carrying TMD1 transgene expression
