# Supplementary material for: Thrombomodulin facilitates melanoma progression via FAK- and ezrin-mediated phenotypic plasticity
Source: J Biomed Sci. 2026 Jan 27;33:14. doi: 10.1186/s12929-026-01217-2 (PMC12849159; doi:10.1186/s12929-026-01217-2)
Supplement: Supplementary file 1 — Supplementary material 1. [file 12929_2026_1217_MOESM1_ESM.docx]

**Title:** Thrombomodulin Facilitates Melanoma Progression via FAK- and Ezrin-Mediated Phenotypic Plasticity

**Other supporting materials for this study include the following:**

**Table S1**

**Figures: S1 to S11**

**Videos: video 1 and video 2**

**Supplementary table**

| Table S1. Antibodies used in the study | |  |  |
| --- | --- | --- | --- |
| antibody | application (dilution) | vender | catalog |
| anti-TM | western blot (1:1000) | Santa Cruz | sc-13164 |
| anti-TM | immunofluorescence staining (1:100) | R&D | AF3947 |
| anti-TM | immunohistochemistry (1:300) | R&D | AF3947 |
| anti-TMD1 | immunohistochemistry (1:100) | in-house |  |
| anti-TMD23 | immunohistochemistry (1:100) | in-house |  |
| anti-hCD31 | immunohistochemistry (1:100) | R&D | AF806 |
| anti-hCD31 | immunohistochemistry (1:100) | abcam | ab28364 |
| anti-mCD31 | immunohistochemistry (1:100) | R&D | AF3628 |
| anti-GAPDH | western blot (1:10000) | Proteintech | HRP-60004 |
| anti-alpha tubulin | western blot (1:20000) | Proteintech | HRP-66031 |
| anti-ezrin | immunofluorescence staining (1:100) | Santa Cruz | sc-20773 |
| anti-ezrin | western blot (1:1000) | Santa Cruz | sc-58758 |
| anti-pT567 ezrin | immunofluorescence staining (1:100) | GeneTex | GTX133868 |
| anti-pY576 FAK | western blot (1:1000) | Santa Cruz | sc-16563-R |
| anti-FAK | western blot (1:1000) | Santa Cruz | sc-558 |
| anti-CD133 | western blot (1:500) | Santa Cruz | sc-365537 |
| anti-Melan-A | immunohistochemistry (1:50) | Santa Cruz | sc-20032 |

**Supplementary Figures**

**
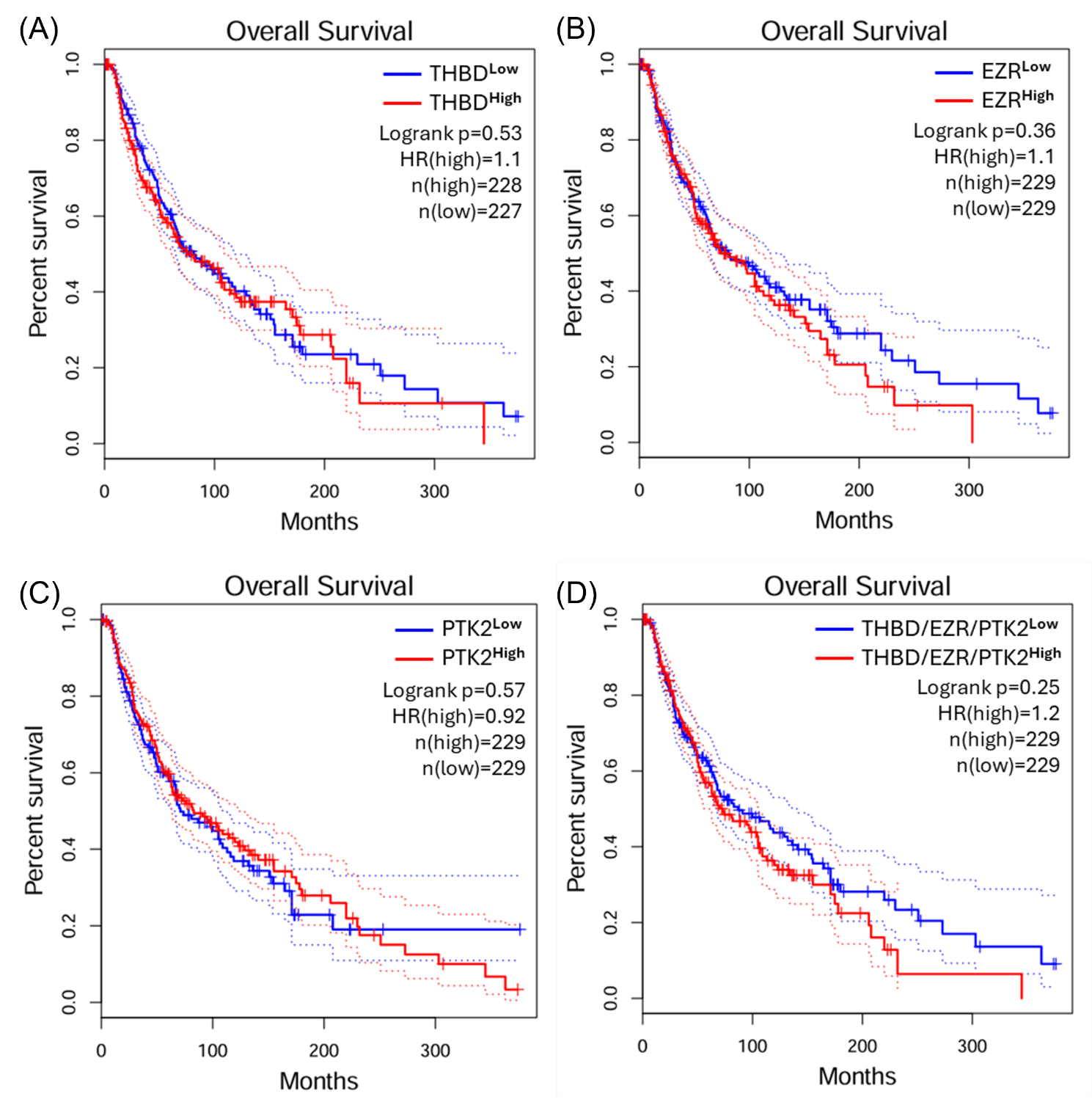
**

Figure S1. Association of specific mRNA expression with overall survival in melanoma patients.

Kaplan–Meier survival analyses were performed using the GEPIA2 online platform to assess the prognostic significance of (A) THBD (TM), (B) EZR (ezrin), (C) PTK2 (FAK), and (D) the combined expression of all three mRNAs in patients with skin cutaneous melanoma (SKCM) from The Cancer Genome Atlas (TCGA) dataset. Patients were stratified into high- and low-expression groups based on the median mRNA expression level.


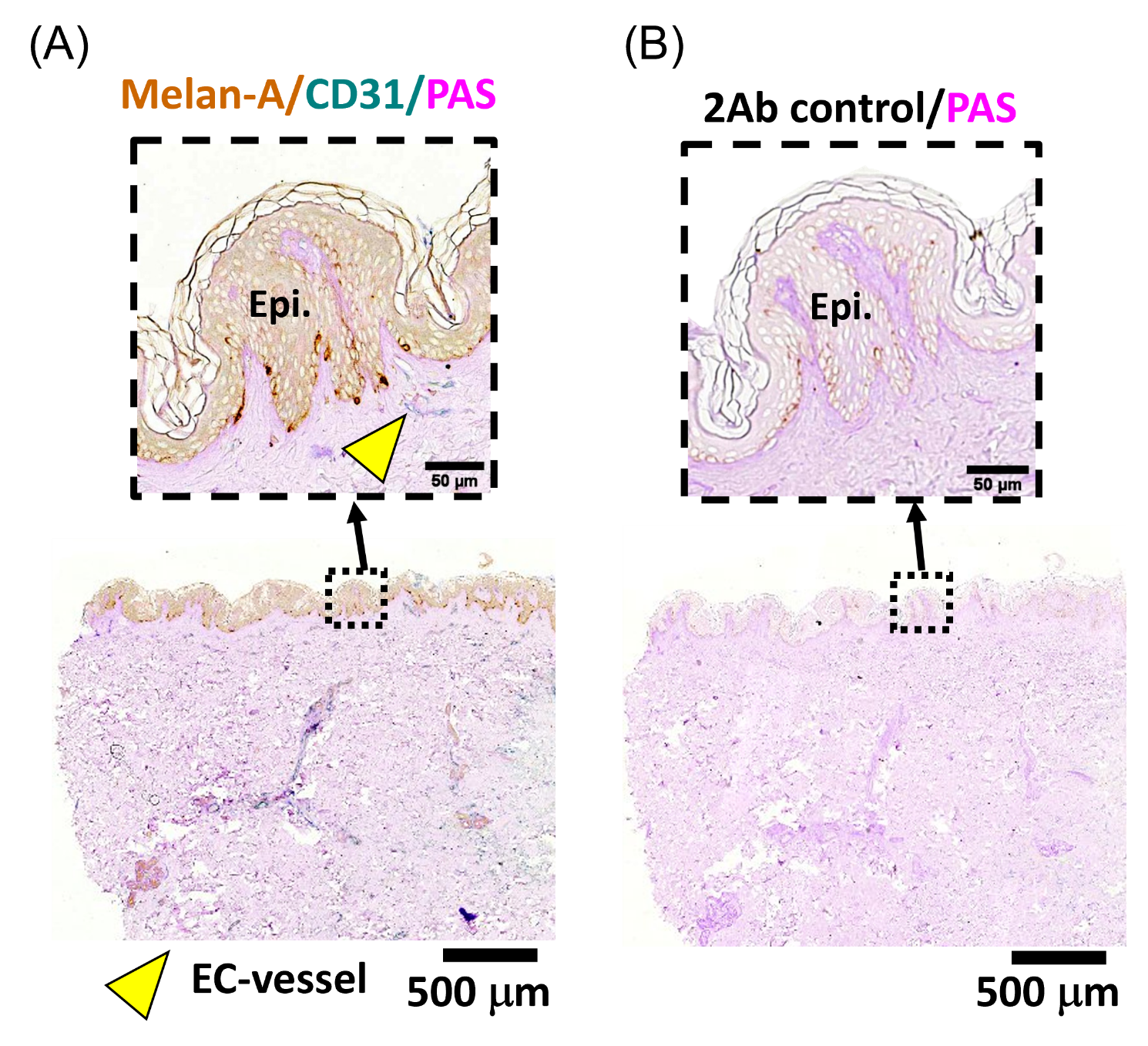


Figure S2. Representative immunohistochemistry of a normal abdominal skin biopsy displaying PAS (magenta) with (A) melan-A (brown) and CD31 primary antibodies (green) or (B) without primary antibody. Epi. indicates the epidermis. The yellow triangle indicates the EC-vessel.


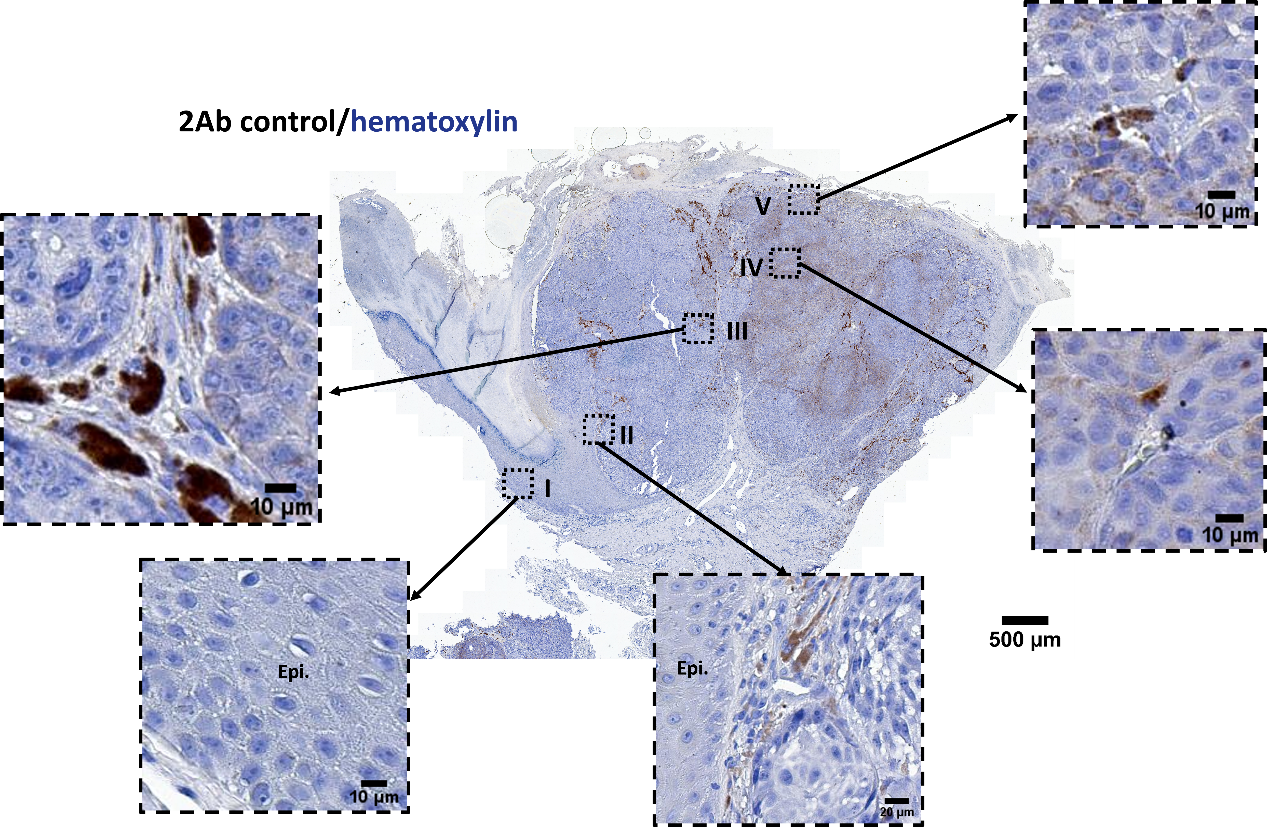


Figure S3. Representative immunohistochemistry without primary antibody images of a skin biopsy from the patient with cutaneous melanoma displaying nucleus (hematoxylin, deep blue-purple). The skin sample was derived from the right sole with a Clark’s level IV melanoma lesion.


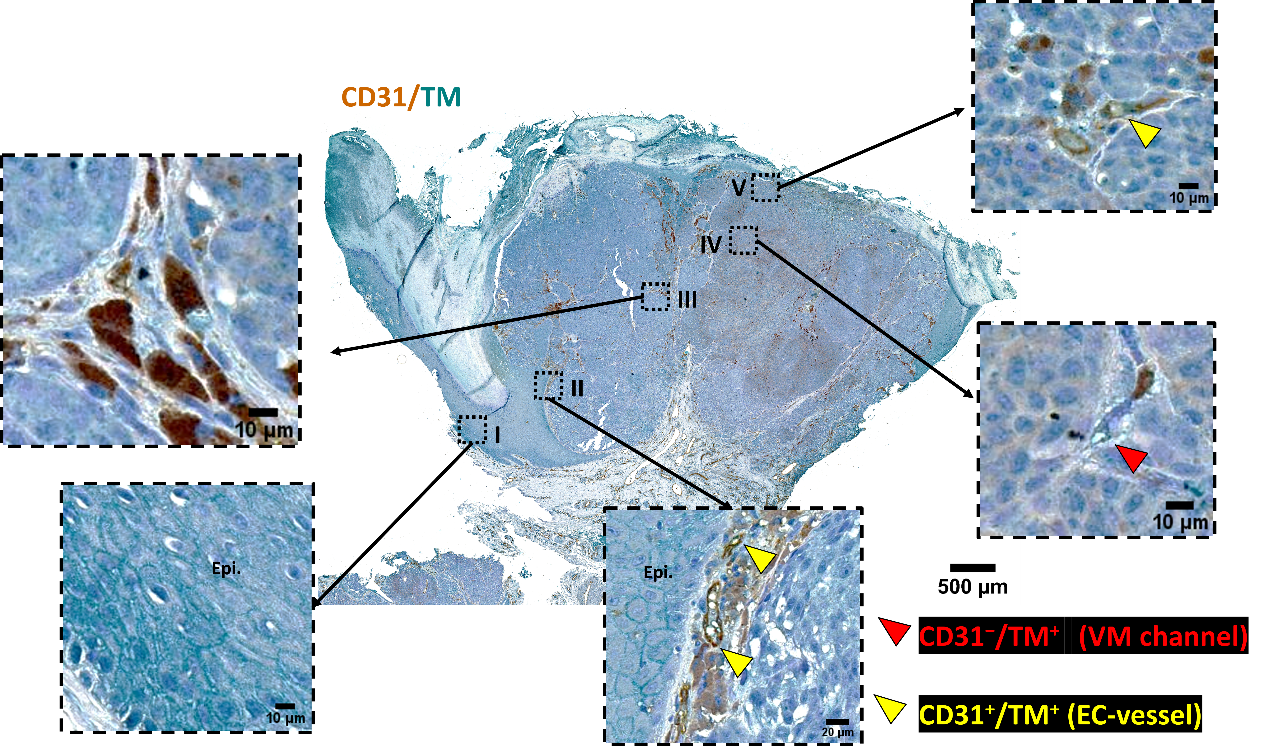


Figure S4. Representative immunohistochemistry images of a skin biopsy from the patient with cutaneous melanoma displaying CD31 (brown), TM (green), and nucleus (hematoxylin, deep blue-purple). The skin sample was derived from the right sole with a Clark’s level IV melanoma lesion. Angiogenesis is mainly composed of endothelial cells which display CD31^+^ and TM^+^ phenotype (yellow arrows). Non-angiogenic vasculatures which refer to vascular mimicry (VM) are constituted of cancer cells that display TM^+^ and CD31^-^ phenotype (red arrows).


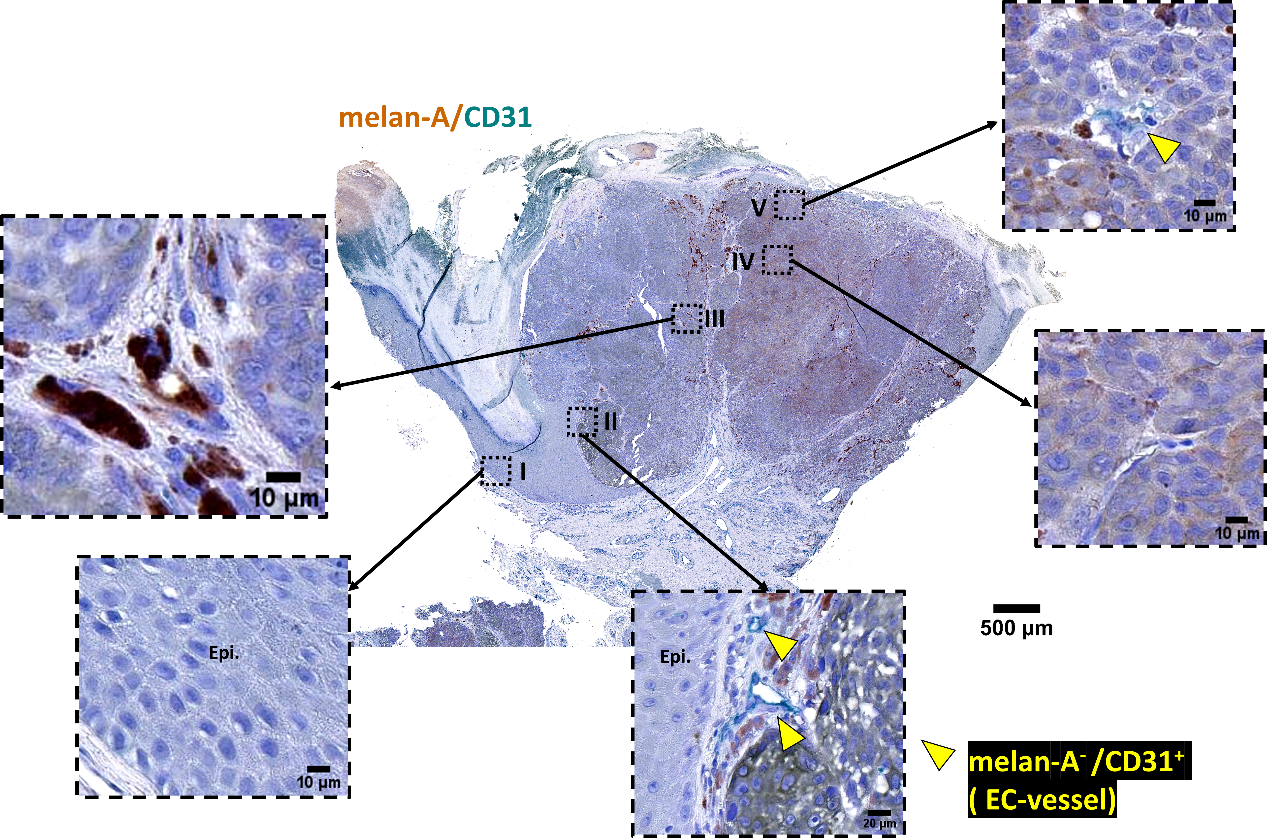


Figure S5. Representative immunohistochemistry images of a skin biopsy from the patient with cutaneous melanoma displaying melan-A (brown), CD31 (green), and nucleus (hematoxylin, deep blue-purple). The skin sample was derived from the right sole with a Clark’s level IV melanoma lesion. Angiogenesis is mainly composed of endothelial cells that display melan-A^-^ and CD31^+^ phenotype (yellow).


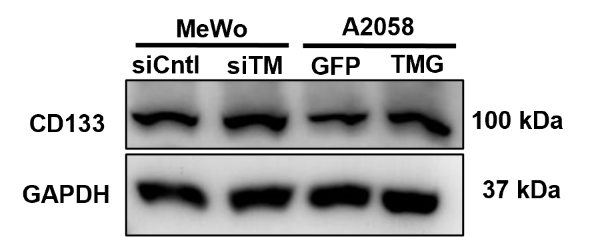


Figure S6. Representative images of western blot of CD133 and GAPDH expression in melanoma cells.


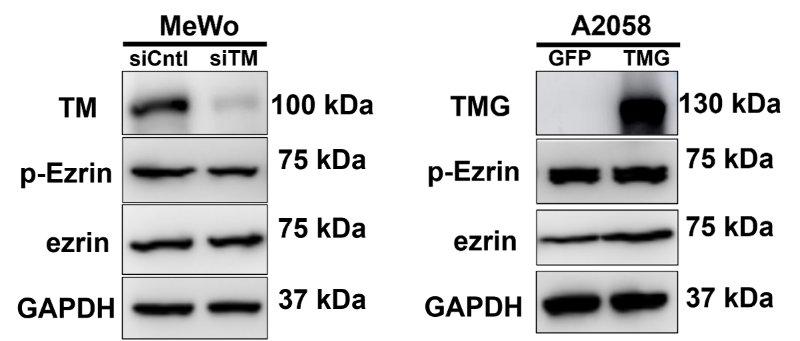


Figure S7. Representative western blot analysis of ezrin expression in melanoma cells, including MeWo cells with siRNA against TM (siTM) or scrambled control (siCntl) and A2058 cells with GFP or TMG.


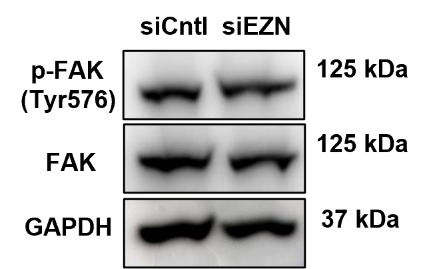


Figure S8. Representative western blot analysis of FAK expression in A2058-TMG cells. The cells were transfected with siRNA targeting ezrin (siEZN) or scrambled control (siCntl) as demonstrated in Figure 5D.


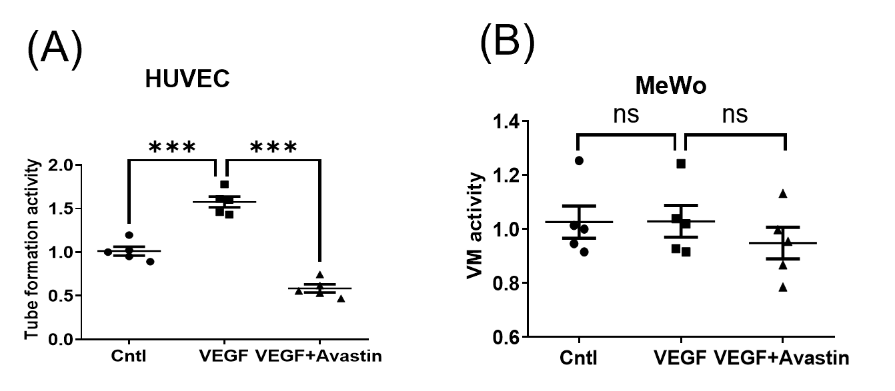


Figure S9. The effect of VEGF treatment in combination with anti-VEGF on the tube formation in (A) HUVECs and (B) MeWo cells on Matrigel. The cells were treated with either VEGF (20 ng/mL) or in combination with anti-VEGF (10 μg/mL; Avastin), and the total tube length was measured. N=5. ***, P< 0.001.


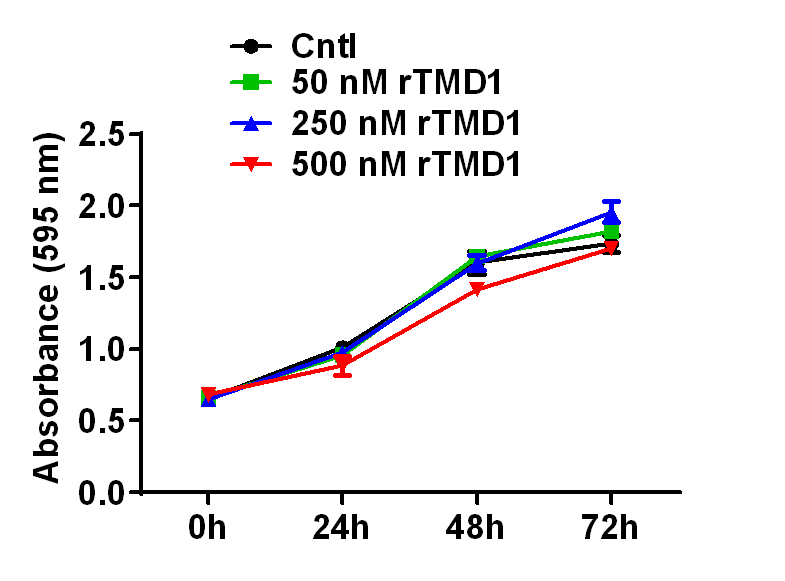


Figure S10. Cell proliferation assay. rTMD1 did not affect the proliferation of MeWo cells. The cell proliferation assay was conducted using the MTT assay. N=6.


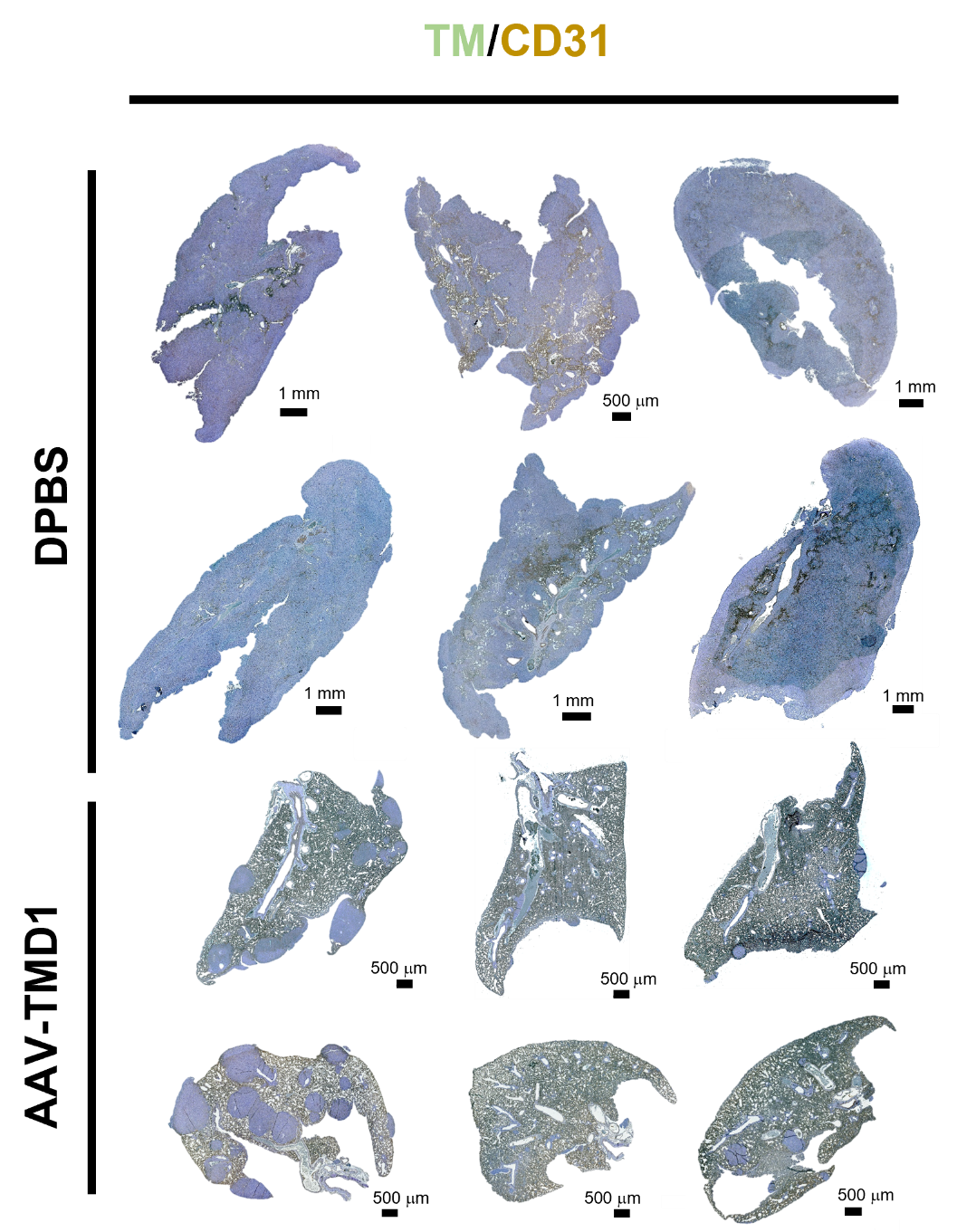


Figure S11. Representative images of CD31 (brown) and TM (green) stains on the mouse lung section from the experimental lung metastasis assay (Figure 7J). Two lung lobes from each mouse were analyzed.

**Video 1 (separate file).** **VM activity of A2058 cell clones on Matrigel.** A time-lapse recording of the GFP channel of tube formation on Matrigel.

**Video 2 (separate file).** **VM activity of A2058 cell clones on Matrigel.** A time-lapse recording of the bright-field channel of tube formation on Matrigel.
